# Associations between widowhood status/duration, depression, and cognitive function among community-dwelling Indians age 60 years or older: Exploration of sex and residential factors

**DOI:** 10.1007/s00127-025-02950-z

**Published:** 2025-06-27

**Authors:** T. Muhammad, Christina X. Mu, Shobhit Srivastava, Vinod Joseph Kannankeril Joseph, Drishti Drishti, Waad Ali, Preeti Pushpalata Zanwar

**Affiliations:** 1 Department of Human Development and Family Studies| Center for Healthy Aging, Pennsylvania State University, University Park, PA 16802 USA; 2 Department of Epidemiology and Biostatistics, University of California, San Francisco, USA; 3 International Institute for Population Sciences, Mumbai, 400088 Maharashtra India; 4 Alma Mater Studiorum - Università di Bologna, Via delle Belle Arti, Bologna, BO Italy; 5 Sultan Qaboos University, Muscat, Oman; 6 Thomas Jefferson University, Philadelphia, PA USA; 7 Texas A&M Health, College Station, TX USA

**Keywords:** Widowhood duration, Depressive symptoms, Cognitive function, Rural-urban, Multigenerational

## Abstract

**Background:**

The death of a spouse is considered one of the most life challenging stressors. Widowhood has a profound influence on health and may increase the risk of depression and poorer cognitive function. Discriminatory practices in India, such as taboos against remarrying, a lack of occupational opportunities, and social support, may lead to differential widowhood experiences, especially among women. This study examined the associations between widowhood status/duration, depression and cognitive function among community-dwelling men and women in India. Considering the unique cultural and societal context in India, this study also sought to examine differences by sex, rural/urban residence, and multigenerational living status.

**Methods:**

We used baseline data from the Longitudinal Aging Study in India. Data were collected between 2017 and 2019. The study sample consisted of 14,691 men and 15,948 women age ≥ 60 years. Depression was measured using the Short Form Composite International Diagnostic Interview and global cognitive functioning was measured using an assessment adapted from the Mini-Mental State Examination and the cognitive module of the United States Health and Retirement Study, and its sister studies. We employed adjusted multivariable logistic and linear regression models to examine the association of widowhood status/duration with the risk of depression, and cognitive function.

**Results:**

Compared to currently married, those widowed within 0–9 years had a higher risk of depression (Men: aOR = 1.65, 95% CI: 1.20, 2.27; Women: aOR = 1.57, 95% CI = 1.25, 1.98) and worse cognitive functioning (Men: *B* = 0.80, 95% CI: 0.30, 1.30; Women: *B* = 0.55, 95% CI = 0.20, 0.91). Among those widowed within 0–9 years, men had a slightly greater risk of worse cognitive functioning than women. As widowhood duration increased, the association between widowhood and worse cognitive functioning was no longer significant among men but remained significant among women. Analyses stratified by rural/urban residence and multigenerational living status and their interactions with widowhood status/duration revealed similar trends. However, the associations between widowhood status/duration and worse cognitive function were more pronounced among women in non-multigenerational households (interaction *p* <.05).

**Conclusions:**

Older adults who were widowed within 0–9 years had a higher risk of depression and worse cognitive functioning. The adverse effects of widowhood on cognition were no longer significant among men but persisted for women with longer widowhood duration. Non-multigenerational households exacerbated the influence of widowhood on the higher risk of depression and worse cognitive functioning, but findings by urban/rural residence were mixed. Future research should explore what other factors moderate widowhood and health relations and examine changes in widowhood duration over time.

**Supplementary Information:**

The online version contains supplementary material available at 10.1007/s00127-025-02950-z.

## Background

India has the largest population in the world and is one of the fastest-growing economies [1]. Globally, there has been a shift toward an aging population and longer life expectancy. The aging trend in the global population does not exhibit gender neutrality, as women constitute the majority of the older population worldwide and in turn, older women disproportionally face higher rates of widowhood [2]. This advantage is due to the gender gap in mortality; that is, although by nature more males are born than females, males have higher mortality across most life stages [3], except during infancy and childhood in the Indian context [4, 5].

Widowhood is considered one of life’s most challenging stressors, and there is evidence that experiences of widowhood increase depression [6, 7]. In most developing countries, women are more likely to surpass men dueseveral factors, including but not limited to men’s tendency to marry women younger and their higher mortality rates. Women are also less likely to remarry after their spouses’ death due to greater societal restrictions on the remarrying and because there are fewer available men in older age groups due to the skewed sex ratio [6–8]. Consequently, older women may remain widowed, whereas men have the opportunity to seek new partners [9–11].

Several studies have found that widowhood has substantial adverse effects on the psychological well-being of older women compared with married women, even several years after widowhood [12–15]. Previous studies have reported that the trajectory of psychological distress and adverse effects due to widowhood appears to follow a “crisis” model, with distress peaking shortly after widowhood and gradually diminishing over time [14, 16]. Similarly, widowhood has been found to have a significant adverse impact on cognitive function, including greater memory decline and an increased risk of Alzheimer’s disease and dementia [17–20].

Importantly, widowhood has severe consequences for the physical, economic, and psychological well-being of older adults, particularly among older women in India, where gender-based discrimination and preferential treatment that are deeply rooted in both cultural traditions and social structures still exist [21–23]. For instance, in addition to societal restrictions on remarrying, some women are forced into exploitative work situations and cannot receive their deceased spouses’ inheritance and land [24]. In addition, discriminatory practices lead to differential widowhood experiences among Indian women, including withdrawal and exclusion from society, which negatively influence daily life. Jain and colleagues [25] found that female widows had worse cognitive functioning, and this effect gradually increased for up to 20 years but plateaued after 20 years. Depression and body mass index were found to be significant mediators in the relationship between widowhood and cognitive functioning [25].

Building on Jain and colleagues’ [25] prior work, this study examines unique societal and residentialfactors that may influence the association between widowhood, depression, and cognitive functioning. This study focuses on differences by sex, living situation, and rural/urban setting. Culturally, multigenerational households in which parents and children live together are commonplace in India, especially after the death of a spouse [26]. Familial support can be an important buffer for reducing loneliness and isolation as well as help families cope as a unit [27, 28]. Additionally, in India, the majority of people live in rural areas, which tend to be more traditional, conservative, and have less access to healthcare resources [29]. These societal and residential factors may moderate the relationship between widowhood and health outcomes.

**Fig. 1:**
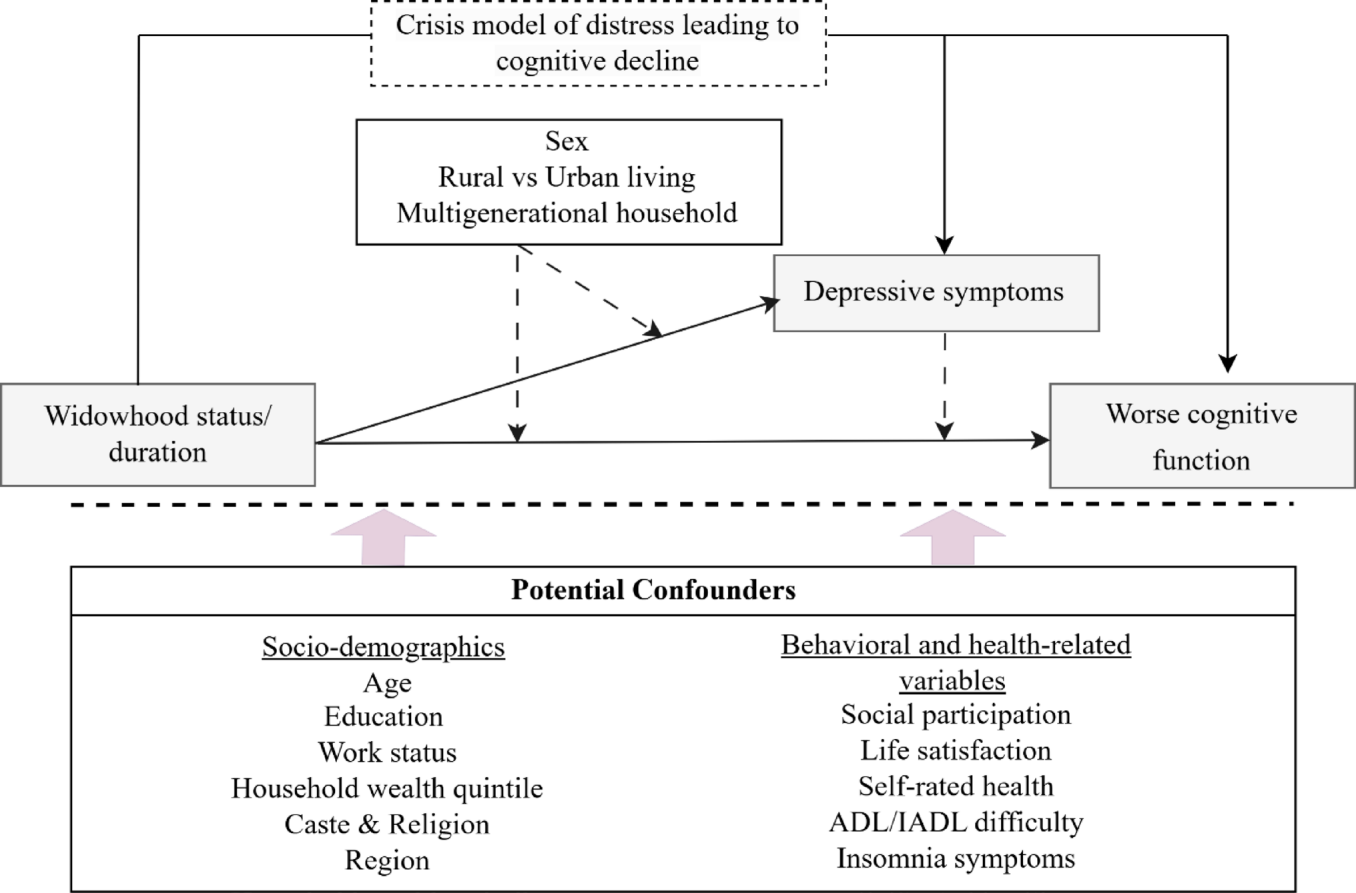
Conceptual research model

This study aimed to examine the associations between widowhood status and duration, depression, and cognitive function in older men and women in India. We hypothesized that longer widowhood duration is associated with a higher risk of depression and worse cognitive function.  . Grounded in the crisis model of distress [30, 31], the framework suggests that bereavement-related stress contributes to cognitive decline, particularly in the presence of psychological distress [32]. Therefore, we also examined moderations by societal and residential factors. We hypothesized that for women, those who are depressed, those residing in rural areas, and those living in non-multigenerational households, the association between widowhood status/duration and poorer outcomes would be exacerbated. Figure 1 presents the conceptual research model.

## Materials and methods

### Data

We used data from the Longitudinal Aging Study in India (LASI) baseline wave (Wave 1) conducted between 2017 and 2019. The survey was a joint undertaking of Harvard T.H. Chan School of Public Health, the International Institute for Population Sciences (IIPS), and the University of Southern California (USC). This nationally representative longitudinal survey collected information on the physical, social, and cognitive well-being of older adults in India, who will be followed up for 25 years. Data from over 72,000 individuals aged 45 years and above, along with their spouses (irrespective of age), were collected across all states and union territories of India. The sample was based on a multistage stratified cluster sample design, including three and four distinct stages of rural and urban area selection, respectively. The survey provides scientific insights and facilitates a harmonized design that helps in comparison with parallel international studies. Further, the details of sample design, survey instruments, fieldwork, data collection and processing, and response rates are publicly available in the LASI report on the LASI website (International Institute for Population Sciences (IIPS) et al., [33]. All participants provided written and oral consent, and the study was approved by the Institutional Review Board of the International Institute for Population Sciences (IIPS), Mumbai, India.

The LASI data included 15,098 older men and 16,366 older women, and after excluding 825 older adults (407 men and 418 women) who were divorced (*n* = 89), separated (*n* = 166), deserted (*n* = 99), in a live-in relationship (romantically committed but not legally married) (*n* = 170) or never-married (*n* = 301), the current analytic study sample consisted of 14,691 men and 15,948 women aged ≥ 60 years. The exclusion criteria were determined in order to focus on the associations between widowhood status (and its duration) and mental and cognitive health among older men and women.

### Variable description

#### Outcome variables

Depression was calculated using the 10-item Short Form Composite International Diagnostic Interview (CIDI-SF) [34], which is a structured diagnostic interview guided by the Diagnostic and Statistical Manual of Mental Disorders that has been validated in epidemiological and cross-cultural studies [35–37]. CIDI-SF items were summed (possible range 0–10), and scores ≥ 3 were categorized as having major depression. The Cronbach’s alpha indicated acceptable reliability (α = 0.68). Depression was a main outcome of the study, but also examined as a moderator between widowhood status/duration and cognitive functioning.

The measure for cognitive functioning was adapted from the Mini-Mental State Examination (MMSE) [38], the cognitive module of the United States Health and Retirement Study (HRS), and its sister studies, the China Health and Retirement Longitudinal Study (CHARLS) and the Mexican Health and Aging Study (MHAS) [39, 40]. These measures have been validated and have been used in previous studies. [41–43]. Five broad cognitive domains were captured: memory, orientation, arithmetic function, executive function, and object naming. Memory was measured using immediate (0–10 points) and delayed (0–10 points) word recall. Orientation was measured using time (0–4 points) and place (0–4 points) measurements. Arithmetic function was measured through backward counting (0–2 points), a serial seven-subtraction task (0–5 points), and a task involving two computations (0–2 points) [39]. Executive function tasks consisted of paper folding (0–3 points) and pentagon drawing (0–1 point). Finally, object naming involved the interviewer pointing at specific objects and asking participants to name them (0–2 points) [40]. A composite sum of the five domains were created and reverse coded, so that higher values indicated worse cognitive functioning (Range = 0–43). Worse cognitive function was treated as a continuous variable.

### Explanatory variable

#### Widowhood status/duration

Our main exploratory variable was widowhood, both status and duration, compared to those currently married. Participants were asked how many years and months ago they became widowed, if applicable. Widowhood duration was rounded to the nearest year.Widowhood status/duration was coded as currently married/not widowed (= 0), widowed within 0–9 years (= 1), widowed within 10–19 years (= 2), and widowed ≥ 20 years ago (= 3). For sensitivity analyses, we also classified widowhood status/duration into currently married/not widowed (= 0), widowed within 0–4 years (= 1), widowed within 5–9 years (= 2), widowed within 10–14 years (= 3), widowed within 15–19 years (= 4), and widowed ≥ 20 years ago (= 5).

### Moderators

Multigenerational living, which included living with children or living with children and others, was coded as a binary variable (yes/no). The place of residence was coded as rural (village) or urban (ward or town), following the Census classification.

### Covariates

The following socio-demographic, behavioural, health-related, and household/community-related variables were considered potential confounders in the relationship between widowhood status/duration and the risk of depression and cognitive functioning, based on existing literature [22, 25, 44, 45].

#### Socio-demographic factors

Participants were asked to specify their sex (men or women), and. was included as a stratification variable. Age was was assessed by asking respondents to report their age in completed years, and was classified into 60–69 years, 70–79 years, and 80 + years. Educational status was coded as no formal education, primary not completed, primary, and secondary and higher. Working status was categorized as: never worked (defined as having not worked for at least three months during their lifetime, including agricultural work, wage work, self-employment, and unpaid family business work, but excluding personal housework regardless of wages), currently not working, currently working, and retired.

#### Behavioural and health-related factors

Social participation was measured using the question, “Are you a member of any of the organizations, religious groups, clubs, or societies?”. This single-item measure, used in large-scale surveys such as the Health and Retirement Study (HRS), does not include follow-up questions on specific organizations, activities, or duration. Responses were coded as ‘*no*’ or ‘*yes*’. Life satisfaction was assessed using the Satisfaction with Life Scale, which has been validated in previous studies [46, 47]. The scale consists of five items: (a) *In most ways*,* my life is close to ideal*; (b) *The conditions of my life are excellent*; (c) *I am satisfied with my life*; (d) *So far*,* I have got the important things I want in life*; and (e) *If I could live my life again*,* I would change almost nothing*. Response options were coded as follows: *strongly disagree* (= 1), *somewhat disagree* (= 2), *slightly disagree* (= 3), *neither agree nor disagree* (= 4), *slightly agree* (= 5), *somewhat agree* (= 6), and *strongly agree* (= 7). A scale was constructed, with scores ranging from to 5–35. We further classified the scale into ‘low satisfaction’ (score of 5–20), ‘medium satisfaction’ (score of 21–25), and ‘high satisfaction’ (score of 26–35).

Self-rated health demonstrated satisfactory construct validity in the Indian context and was consistently correlated with various health outcomes [48, 49]. Following previous studies [50, 51], we dichotomized self-rated health into two categories for analytical purposes and coded as “good”, which included *excellent*, *very good* and *good* whereas “poor” included *fair* and *poor*. Difficulty in activities of daily living (ADL) was coded as no (= 0, reference category) or yes (= 1). ADL refers to routine daily self-care tasks that are typically required for individual well-being and maintenance, including but not limited to the ability to perform movements in bed, changing positions from a sitting to a standing posture, engaging in feeding, bathing, dressing, grooming, and personal hygiene. Difficulty with instrumental ADL (IADL) was coded as “no” or “yes”. Respondents were asked if they had any difficulties that were expected to last more than three months, such as preparing a hot meal, shopping for groceries, making a telephone call, taking medications, doing work around the house or garden, managing money (such as paying bills and keeping track of expenses), and getting around or finding an address in an unfamiliar place. The ADL and IADL tools used to assess functional ability have been validated across various settings [52]. Additionally, insomnia symptoms, known to affect depression and cognitive performance [53, 54], were assessed using the Jenkins Sleep Scale (JSS-4) [55], a brief questionnaire designed to identify sleep difficulties over the past month and is widely used in epidemiological studies [56]. It includes four items related to trouble falling asleep, waking during the night, early waking, and feeling unrested during the day, with response options ranging from “never” to “frequently.” Insomnia symptoms were coded as 1 if any symptom was reported “occasionally” or “frequently”. Insomnia symptoms were treated as a binary variable (0 = no insomnia symptoms, 1 = one or more insomnia symptoms).

#### Household/Community-related factors

The monthly per capita expenditure (MPCE) quintile was assessed using household consumption data. Sets of 11 and 29 questions were used to survey expenditures on food and non-food items, respectively, in participants’ households. Food expenditure was collected based on a reference period of seven days, and non-food expenditures were collected based on reference periods of 30 days and 365 days. Food and non-food expenditures were standardized to the 30-day reference period. MPCE was computed and used as a summary measure of consumption. The variable was then divided into five quintiles from the poorest to the richest.

The caste system is a social hierarchy based on family lines, occupation, and economic status [57]. Although the influence of the caste system has waned over the years, societal pressures still reinforce class disparities [58]. Religion is another important cultural component. India is a religious country with the majority identifying Hinduism, followed by Islam, Christianity, and other religions (e.g., Sikhism, Buddhism, and Jainism) [59]. Caste was recorded as scheduled tribe, scheduled caste, other backward classes, and others. The scheduled caste includes a group of the population that is socially segregated and financially/economically by their low status, as per the Hindu caste hierarchy. The scheduled tribes and castes constitute two of the most marginalized socioeconomic groups in India. Other backward classes is an official Indian government term for socially and economically disadvantaged groups, often associated with affirmative action policies related to employment and education and for social justice [60]. The “other” caste category is identified as people having higher social status [61]. Religion was coded as Hindu, Muslim, Christian, and Others. The region was coded as North, Central, East, Northeast, West, and South.

### Statistical analysis

Descriptive statistics and cross-tabulation were used to summarize main features of the sample. The chi-square test was used to assess the statistical significance of depression, whereas ANOVA was applied to examine differences in cognitive function. Subsequently, adjusted binary logistic and linear regression models were used to examine the associations between widowhood status/duration on depression and worse cognitive function. Stratified analyses were conducted to examine the differences in the observed associations by sex, place of residence, and multigenerational living. Additionally, interaction models were used to assess whether the association between widowhood status/duration and cognition was influenced by depression status, and whether the relationships between widowhood status/duration, depression, and worse cognitive function, were modified by place of residence and multigenerational living. We reported adjusted estimates (adjusted odds ratios and beta coefficients with 95% confidence intervals) after controlling for selected covariates and used margin plots to visualize the interaction effects. The variance inflation factor was estimated to check for multicollinearity; there was no evidence of multicollinearity among the variables included in this analysis. Individual weights were utilized to ensure that the study’s estimates were nationally representative. The analysis was performed using STATA version 18.

## Results

Table 1 presents the sample characteristics. Slightly more than half of the sample were between 60 and 69 years of age (58% men, 59% women). Most men in the sample were currently married (84% men, 46% women). A large proportion (38% men, 73% women) of the sample had no formal education, reflecting limited educational opportunities for older generations in India and in women in particular; 32% men and 11% women had secondary or higher education. Nearly 69% of the men and 70% of the women lived in multigenerational households. Older widows living with their children or in multigenerational households are normalized in Indian culture compared to Western countries, and this practice is attributed to the cultural roots and beliefs of Dharma, in which older parents are well respected and cared for [26]. A large proportion of the sample were Hindus (82.02% men and 82.34% women), followed by Muslims (11.81% men and 10.95% women), closely mirroring the population’s religious composition.

**Table 1:** Socio-demographic and health characteristics of older men and women in India, LASI, 2017-2019 (n=30,639)

Background characteristic	Men (*n* = 14,691) *n* (%)	Women (*n* = 15,948) *n* (%)
*Widowhood status/duration*		
Currently married	12,398 (83.6)	7522 (46.47)
Widows (0–9 years)	1132 (8.32)	2822 (18.9)
Widows (10–19 years)	598 (4.15)	2398 (15.63)
Widows (≥ 20 years)	476 (3.94)	2720 (19.01)
*Age*		
60–69 years	8727 (57.85)	9724 (58.95)
70–79 years	4430 (31.3)	4455 (29.42)
*≥* 80 years	1534 (10.85)	1769 (11.63)
*Education*		
No formal education	5300 (38.43)	11,176 (72.84)
Primary not completed	2116 (14.45)	1546 (8.62)
Primary	2247 (14.85)	1434 (7.85)
Secondary/higher	5027 (32.26)	1792 (10.68)
*Multigenerational living*		
Yes	10,345 (68.65)	11,258 (69.53)
No	4346 (31.35)	4690 (30.47)
*Working status*		
Never worked	707 (3.68)	7857 (47.05)
Currently not working	5805 (40.76)	4903 (32.63)
Currently working	5899 (42.17)	2836 (18.54)
Retired	2280 (13.39)	352 (1.78)
*Social participation*		
No	13,185 (94.01)	14,951 (96.46)
Yes	1260 (5.99)	804 (3.54)
Life satisfaction*		
Low	4129 (30.04)	4871 (33.52)
Medium	3329 (22.4)	3724 (22.3)
High	6713 (47.56)	6823 (44.18)
Self-rated health*		
Good	11,418 (78.01)	11,717 (74.18)
Poor	2970 (21.99)	3900 (25.82)
Difficulty with ADL*		
No	11,969 (79.18)	12,034 (73.61)
Yes	2639 (20.82)	3874 (26.39)
Difficulty with IADL*		
No	9508 (61.46)	7511 (43.1)
Yes	5089 (38.54)	8371 (56.9)
*Insomnia symptoms*		
No	9509 (62.69)	9009 (55.27)
Yes	5116 (37.31)	6905 (44.73)
*MPCE quintile*		
Poorest	2936 (20.6)	3360 (22.48)
Poorer	2992 (21.41)	3326 (22.03)
Middle	2979 (21.57)	3270 (20.4)
Richer	2923 (19.32)	3107 (19.24)
Richest	2861 (17.1)	2885 (15.85)
*Religion*		
Hindu	10,780 (82.02)	11,681 (82.34)
Muslim	1770 (11.81)	1893 (10.95)
Christian	1416 (2.51)	1589 (3.08)
Others	725 (3.66)	785 (3.63)
*Caste*		
Scheduled Caste	2403 (18.97)	2643 (19.06)
Scheduled Tribe	2367 (7.76)	2625 (8.49)
Other Backward Classes	5587 (45.56)	5934 (44.46)
Others	4334 (27.71)	4746 (27.99)
*Place of residence*		
Rural	9841 (72.00)	10,435 (69.39)
Urban	4850 (28.00)	5513 (30.61)
*Region*		
North	2734 (12.36)	2987 (12.98)
Central	2098 (22.48)	2074 (19.7)
East	2825 (24.85)	2854 (23)
Northeast	1734 (2.93)	1883 (3.02)
West	3374 (20.83)	3856 (23.48)
South	1926 (16.54)	2294 (17.82)

LASI, Longitudinal Aging Study in India; n, Unweighted sample; %, Percentages weighted to account for the complex sample design; ADL, Activities of daily living; IADL, Instrumental activities of daily living; MPCE, Monthly per capita consumption expenditure; Other Backward Classes refer to groups identified by the Indian government as socially and economically disadvantaged for the purpose of affirmative action policies; * p < .05

Table 2 shows the prevalence of depression and the mean score of worse cognitive function among older men and women according to widowhood status/duration and other background characteristics. Older adults who were widowed for 0–9 years had higher rates of depression (12% men and 13% women) than those who were currently married (7% men and 6% women) or widowed for ≥ 20 years (5% men and 7% women). Older adults who were widowed for ≥ 20 years had worse cognitive functioning compared to those who were currently married.

**Table 2:** Prevalence of depression and mean score of worse cognitive function of older men and women by background characteristics in India, LASI, 201–2019

Background characteristic	Men				Women			
	Depression, %	*p*-value	Worse cognitive function, mean (SD)	*p*-value	Depression, %	*p*-value	Worse cognitive function, mean (SD)	*p*-value
*Widowhood status/duration*		<.001		< .001		<.001		< .001
Currently married	7.21		19.48 (6.12)		8.67		23.16 (6.38)	
Widows (0–9 years)	12.14		21.18 (6.29)		12.73		24.68 (6.39)	
Widows (10–19 years)	6.97		20.92 (6.45)		9.88		24.27 (6.42)	
Widows (≥ 20 years)	5.43		22.14 (6.38)		9.48		25.24 (6.50)	
*Age*		.317		< .001		.003		< .001
60–69 years	7.67		18.94 (5.92)		9.14		23.03 (6.34)	
70–79 years	6.67		20.64 (6.19)		10.03		25.12 (6.21)	
*≥* 80 years	9.30		22.43 (6.73)		12.27		27.42 (6.29)	
*Education*		.043		< .001		<.001		< .001
No formal education	7.75		23.88 (5.6)		10.48		26.23 (5.31)	
Primary not completed	9.49		20.51 (5.35)		9.83		22.6 (5.56)	
Primary	7.84		18.64 (4.93)		8.57		19.37 (5.3)	
Secondary/higher	6.25		15.62 (4.67)		5.5		16.23 (4.9)	
*Multigenerational living*		.48		.004		<.001		.038
Yes	7.30		19.57 (6.17)		8.79		23.85 (6.45)	
No	8.09		20.32 (6.28)		11.93		24.42 (6.6)	
*Working status*		<.001		< .001		<.001		< .001
Never worked	9.50		20.47 (6.87)		7.53		23.21 (6.64)	
Currently not working	8.97		20.96 (6.3)		11.88		25.48 (6.14)	
Currently working	6.22		19.82 (5.92)		11.27		24.36 (5.81)	
Retired	6.99		16.38 (5.26)		12.61		17.48 (6.84)	
*Social participation*		.003		< .001		.107		< .001
No	7.72		19.97 (6.24)		9.75		24.13 (6.48)	
Yes	4.46		17.21 (5.43)		9.43		21.35 (6.39)	
*Life satisfaction*		<.001		< .001		<.001		< .001
Low	12.15		20.88 (6.14)		13.98		25.49 (6.09)	
Medium	7.64		20.05 (6.22)		8		24.15 (6.37)	
High	4.54		19.01 (6.13)		7.33		22.93 (6.68)	
*Self-rated health*		<.001		< .001		<.001		< .001
Good	5.42		19.4 (6.19)		6.94		23.65 (6.49)	
Poor	15.06		21.27 (6.06)		17.78		25.2 (6.4)	
*Difficulty in ADL*		<.001		< .001		<.001		< .001
No	5.79		19.4 (6.09)		7.56		23.46 (6.42)	
Yes	14.52		21.47 (6.42)		16.12		25.76 (6.55)	
*Difficulty in IADL*		<.001		< .001		<.001		< .001
No	5.33		18.65 (5.91)		5.95		22.59 (6.56)	
Yes	11.19		21.82 (6.27)		12.66		25.2 (6.14)	
*Insomnia symptoms*		<.001		< .001		<.001		< .001
No	4.82		19.46 (6.22)		5.74		23.51 (6.63)	
Yes	12.12		20.37 (6.1)		14.69		24.69 (6.26)	
*MPCE quintile*		.019		< .001		.001		< .001
Poorest	7.52		20.94 (6.37)		10.05		25.57 (6.01)	
Poorer	5.89		20.5 (6.14)		9.83		24.65 (6.26)	
Middle	7.73		19.5 (6.12)		8.49		23.97 (6.4)	
Richer	7.69		19.48 (6.01)		9.71		23.11 (6.45)	
Richest	9.24		18.35 (5.97)		10.82		22.39 (6.82)	
*Religion*		<.001		< .001		<.001		< .001
Hindu	7.65		19.68 (6.24)		9.51		23.98 (6.48)	
Muslim	7.50		20.09 (5.79)		11.53		24.89 (6.02)	
Christian	3.40		20.67 (6.17)		10.43		22.59 (6.89)	
Others	8.04		20.85 (6.39)		9.00		23.66 (6.66)	
*Caste*		<.001		< .001		<.001		< .001
Scheduled Caste	9.76		21.21 (6.04)		10.3		25.74 (5.75)	
Scheduled Tribe	4.21		22.56 (6.37)		5.59		26.37 (6.32)	
Other Backward Classes	7.63		19.68 (6.14)		10.82		23.68 (6.43)	
Others	6.77		18.37 (5.84)		8.88		22.88 (6.64)	
*Place of residence*		.001		< .001		<.001		< .001
Rural	8.05		20.78 (6.15)		11.19		25.43 (5.86)	
Urban	6.16		17.24 (5.6)		6.43		21.06 (6.68)	
Region		<.001		< .001		<.001		< .001
North	6.85		19.52 (5.94)		6.74		24.59 (6.25)	
Central	11.89		19.99 (6.17)		17.37		24.76 (5.78)	
East	7.37		19.96 (6.15)		9.18		24.7 (6.18)	
Northeast	4.69		18.97 (6.44)		6.61		23.71 (6.64)	
West	5.18		19.69 (6.44)		6.46		22.3 (6.95)	
South	5.72		19.73 (5.92)		9.19		24.26 (6.49)	
Total sample	7.54		19.79 (6.2)		9.74		24.02 (6.49)	

LASI, Longitudinal Aging Study in India; p-values are based on Chi-square test for depression and ANOVA for worse cognitive function; ADL: Activities of daily living; IADL: Instrumental activities of daily living, MPCE, Monthly per capita consumption expenditure; Other Backward Classes refer to groups identified by the Indian government as socially and economically disadvantaged for the purpose of affirmative action policies

Table 3 presents the results of the logistic regression analyses with depression and linear regression analyses with cognitive functioning. Compared to those who were currently married, older adults who were widowed for 0–9 years had higher odds of depression (men: aOR = 1.65, 95% CI: 1.20, 2.27; women: aOR: 1.57, CI: 1.25, 1.98) and worse cognitive function (men: *B* = 0.80, 95% CI: 0.30, 1.30; women: *B* = 0.55, 95% CI: 0.20, 0.91). Additionally, women who were widowed for 10–19 years (*B* = 0.62, 95% CI: 0.10, 1.14) and ≥ 20 years (*B* = 0.91, 95% CI: 0.45, 1.36) had worse cognitive function compared with women who were currently married.

**Table 3:** Multivariate logistic and linear regression estimates of depression and worse cognitive function by widowhood status/duration (10-year groups) and other background characteristics among older men and women in India, LASI, 2017-2019

Background characteristic	Men		Women	
	Depression, OR (95%CI)	Worse cognitive function, β (95%CI)	Depression, OR (95%CI)	Worse cognitive function, β (95%CI)
*Widowhood status/duration*				
Currently married	Ref.	Ref.	Ref.	Ref.
Widows (0–9 years)	1.65** (1.20, 2.27)	0.80** (0.30, 1.30)	1.57*** (1.25, 1.98)	0.55** (0.20, 0.91)
Widows (10–19 years)	0.89 (0.53, 1.50)	−0.07 (−0.72, 0.59)	1.15 (0.84, 1.59)	0.62* (0.10, 1.14)
Widows (≥ 20 years)	0.66 (0.39, 1.10)	0.67 (−0.40, 1.74)	1.10 (0.84, 1.43)	0.91*** (0.45, 1.36)
*Age*				
60–69 years	Ref.	Ref.	Ref.	Ref.
70–79 years	0.66*** (0.53, 0.82)	0.99*** (0.65, 1.33)	0.92 (0.75, 1.14)	1.19*** (0.83, 1.55)
≥ 80 years	0.70 (0.44, 1.10)	1.91*** (1.29, 2.52)	0.97 (0.70, 1.34)	2.57*** (1.99, 3.15)
*Education*				
No formal education	Ref.	Ref.	Ref.	Ref.
Primary not completed	1.25 (0.88, 1.78)	−3.32*** (−3.80, −2.84)	1.24 (0.90, 1.71)	−3.16*** (−3.64, −2.69)
Primary	1.13 (0.86, 1.50)	−4.64*** (−5.08, −4.19)	1.30 (0.95, 1.80)	−5.55*** (−6.12, −4.98)
Secondary/higher	1.05 (0.80, 1.37)	−6.87*** (−7.24, −6.49)	1.06 (0.71, 1.57)	−7.88*** (−8.52, −7.24)
*Multigenerational living*				
Yes	Ref.	Ref.	Ref.	Ref.
No	1.03 (0.82, 1.29)	0.23 (−0.11, 0.56)	1.29* (1.05, 1.56)	0.39* (0.07, 0.71)
*Working status*				
Never worked	Ref.	Ref.	Ref.	Ref.
Currently not working	0.94 (0.55, 1.59)	−0.40 (−1.18, 0.38)	1.48*** (1.18, 1.85)	−0.30 (−0.64, 0.04)
Currently working	0.88 (0.52, 1.49)	−0.47 (−1.25, 0.31)	1.78*** (1.36, 2.35)	−0.81*** (−1.20, −0.42)
Retired	0.93 (0.52, 1.65)	−1.13** (−1.94, −0.31)	2.02* (1.11, 3.65)	−2.36*** (−3.26, −1.47)
*Social participation*				
No	Ref.	Ref.	Ref.	Ref.
Yes	0.76 (0.49, 1.17)	−0.56** (−0.95, −0.17)	1.11 (0.72, 1.73)	−0.83** (−1.39, −0.26)
*Life satisfaction*				
Low	Ref.	Ref.	Ref.	Ref.
Medium	0.61*** (0.46, 0.82)	−0.32 (−0.72, 0.07)	0.53*** (0.43, 0.67)	−0.73*** (−1.11, −0.36)
High	0.43*** (0.34, 0.54)	−0.45* (−0.81, −0.10)	0.57*** (0.46, 0.72)	−0.88*** (−1.20, −0.55)
Self-rated health				
Good	Ref.	Ref.	Ref.	Ref.
Poor	2.21*** (1.77, 2.75)	0.85*** (0.50, 1.21)	2.12*** (1.74, 2.58)	0.53** (0.20, 0.86)
*Difficulty with ADL*				
No	Ref.	Ref.	Ref.	Ref.
Yes	1.71*** (1.28, 2.30)	0.37 (−0.08, 0.82)	1.44*** (1.17, 1.77)	0.44* (0.06, 0.81)
*Difficulty with IADL*				
No	Ref.	Ref.	Ref.	Ref.
Yes	1.53** (1.15, 2.03)	1.22*** (0.85, 1.59)	1.68*** (1.36, 2.08)	1.02*** (0.69, 1.35)
*Insomnia symptoms*				
No	Ref.	Ref.	Ref.	Ref.
Yes	1.87*** (1.53, 2.29)	0.06 (−0.24, 0.37)	2.09*** (1.73, 2.52)	0.05 (−0.23, 0.34)
*MPCE quintile*				
Poorest	Ref.	Ref.	Ref.	Ref.
Poorer	0.80 (0.59, 1.08)	0.13 (−0.29, 0.55)	1.02 (0.79, 1.32)	−0.32 (−0.73, 0.10)
Middle	1.20 (0.84, 1.72)	−0.32 (−0.75, 0.12)	0.92 (0.68, 1.24)	−0.63** (−1.05, −0.21)
Richer	1.27 (0.92, 1.74)	−0.29 (−0.75, 0.17)	1.12 (0.84, 1.49)	−0.68** (−1.14, −0.23)
Richest	1.47* (1.07, 2.02)	−0.56* (−1.03, −0.09)	1.29 (0.98, 1.70)	−0.86** (−1.40, −0.33)
*Religion*				
Hindu	Ref.	Ref.	Ref.	Ref.
Muslim	0.92 (0.68, 1.24)	−0.32 (−0.82, 0.19)	1.11 (0.79, 1.55)	0.22 (−0.21, 0.64)
Christian	0.64 (0.26, 1.58)	0.56 (−0.23, 1.35)	2.09** (1.27, 3.45)	−0.20 (−0.95, 0.54)
Others	0.96 (0.60, 1.54)	0.44 (−0.17, 1.06)	1.24 (0.80, 1.93)	−0.59 (−1.19, 0.00)
*Caste*				
Scheduled Caste	Ref.	Ref.	Ref.	Ref.
Scheduled Tribe	0.50** (0.33, 0.77)	1.17*** (0.53, 1.81)	0.55** (0.37, 0.81)	0.75** (0.21, 1.30)
Other Backward Classes	0.92 (0.70, 1.22)	−0.42* (−0.82, −0.02)	1.45** (1.13, 1.86)	−0.57** (−0.97, −0.18)
Others	0.84 (0.63, 1.12)	−0.25 (−0.68, 0.18)	1.14 (0.85, 1.54)	−0.55* (−0.97, −0.13)
*Place of residence*				
Rural	Ref.	Ref.	Ref.	Ref.
Urban	0.99 (0.78, 1.26)	−1.19*** (−1.52, −0.85)	0.77* (0.60, 0.99)	−1.43*** (−1.77, −1.09)
*Region*				
North	Ref.	Ref.	Ref.	Ref.
Central	1.91*** (1.42, 2.56)	−0.09 (−0.55, 0.37)	2.68*** (2.01, 3.58)	−0.49* (−0.88, −0.10)
East	0.95 (0.70, 1.29)	−0.11 (−0.50, 0.28)	1.03 (0.78, 1.36)	−0.43* (−0.83, −0.03)
Northeast	0.98 (0.57, 1.67)	−0.27 (−0.82, 0.28)	0.98 (0.65, 1.48)	−0.40 (−0.95, 0.14)
West	0.57** (0.40, 0.80)	−0.15 (−0.58, 0.29)	0.56*** (0.40, 0.77)	−1.22*** (−1.69, −0.75)
South	0.94 (0.65, 1.37)	1.05*** (0.60, 1.51)	1.26 (0.92, 1.73)	0.97*** (0.54, 1.41)

LASI, Longitudinal Aging Study in India; OR: odds ratio adjusted for all the selected covariates; β: beta coefficients adjusted for all the selected covariates; ADL: Activities of daily living; IADL: Instrumental activities of daily living; Ref: Reference; AOR: Adjusted Odds Ratio; *p<.05, **p<.01, ***p<.001; CI: Confidence interval; MPCE: Monthly per capita consumption expenditure; Other Backward Classes refer to groups identified by the Indian government as socially and economically disadvantaged for the purpose of affirmative action policies

**Fig. 2:**
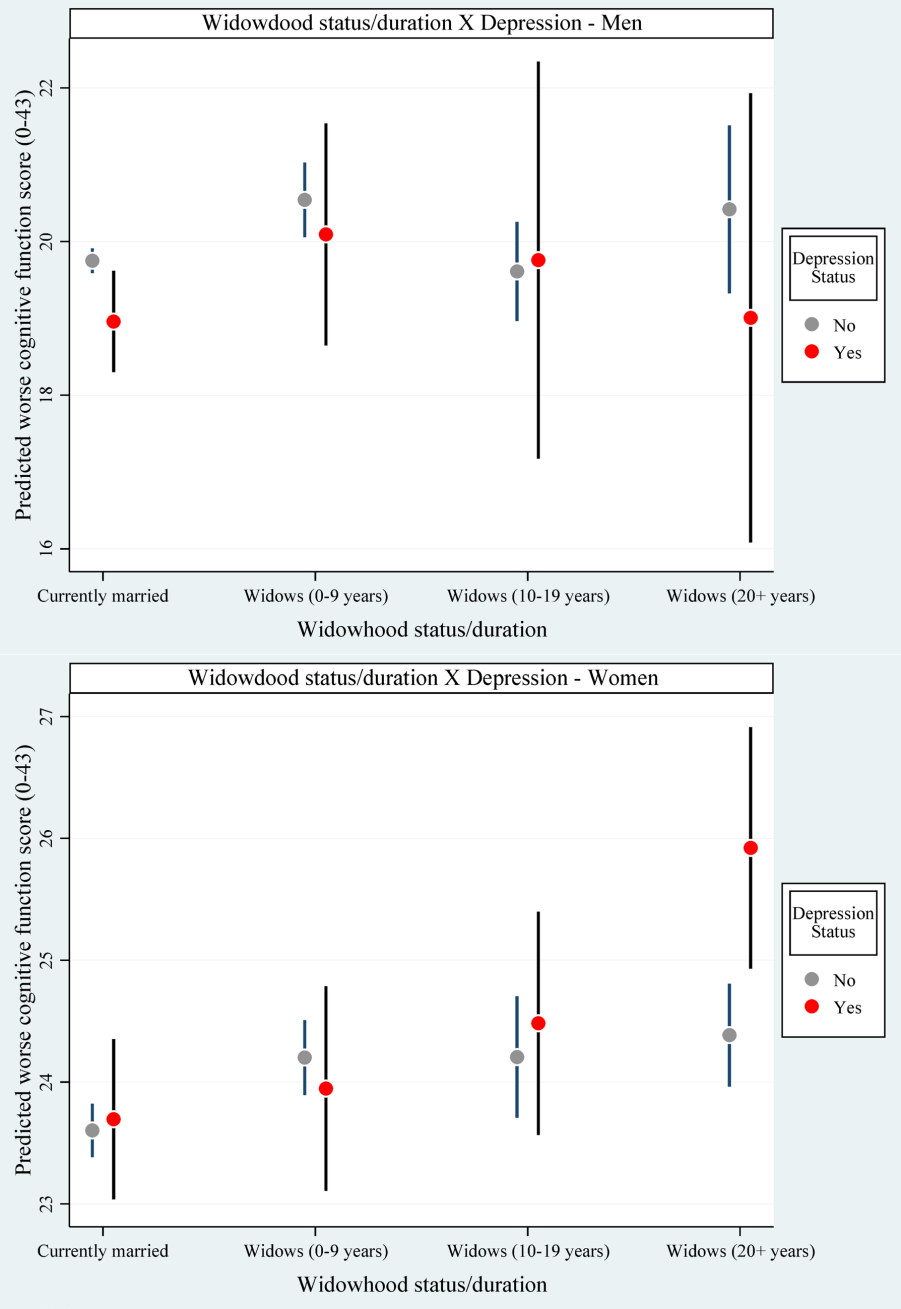
Interaction effect of widowhood status/duration and depression on cognitive function among men and women (adjusted for all the selected covariates)

Figure 2 presents the interaction effect of widowhood status/duration and depression on worse cognitive function among men and women. Older women who were widowed for ≥ 20 years and had depression had higher levels of worse cognitive function than women who were widowed for ≥ 20 years and had no depression (interaction *p*-value =.024).

### Sensitivity analysis

Sensitivity analyses were conducted to check whether the associations were consistent across 5-year groups of widowhood duration. Table S1 presents the results, which remained consistent across 5-year widowhood duration groups. Compared to those currently married, those who were widowed within 0–4 years had higher odds of depression (men: aOR = 2.47, 95% CI: 1.74, 3.53; women: aOR: 1.92, CI: 1.47, 2.51) and worse cognitive function (men: *B* = 0.80, 95% CI: 0.20, 1.41; women: *B* = 0.56, 95% CI: 0.10, 1.01). The association between widowhood/status duration and worse cognitive function was significant among women who had longer widowhood duration (women 5–9 years widowed: *B* = 0.54, 95% CI: 0.09, 1.00; women 10–14 years widowed: *B* = 0.86, 95% CI: 0.15, 1.56; women ≥ 20 years widowed: *B* = 0.90, 95% CI: 0.44, 1.36).

Figure S1 presents the interactions between widowhood status/duration (5-year groups), and depression on worse cognitive function. The results remained consistent with the main analysis that showed that women who were widowed for ≥ 20 years and had depression had more pronounced worse cognitive function than their peers with no depression (interaction *p*-value <.010).

### Supplemental analyses

Tables S2a and S2b display analyses stratified by rural/urban residence for men and women, respectively. Trends were mostly consistent with the main analyses. Among men, those who were widowed for 0–9 years had a higher risk of depression compared to those who were currently married, and this association was more pronounced in those living in urban regions (rural: aOR = 1.59, 95% CI: 1.10, 2.31; urban: aOR: 1.89, CI: 1.03, 3.45). Compared to currently married men, men living in rural regions who were recently widowed within 0–9 years (*B* = 0.82, 95% CI: 0.23, 1.41) and men living in urban regions who were widowed ≥ 20 years (*B* = 1.43, 95% CI: 0.24, 2.62) had worse cognitive functioning. Among women who were widowed within 0–9 years, those living in urban regions had higher odds of depression (rural: aOR = 1.55, 95% CI: 1.19, 2.02; urban: aOR: 1.71, CI: 1.10, 2.67) and worse cognitive functioning compared to those living in rural regions (rural: *B* = 0.47, 95% CI: 0.04, 0.90; urban: *B* = 0.74, 95% CI: 0.10, 1.37).

Tables S3a and S3b display the analyses stratified by multigenerational living status for men and women, respectively. For men living in multigenerational households, those who were recent widows within 0–9 years and those who were widowed for ≥ 20 years had worse cognitive functioning compared to currently married men (men widowed 0–9 years: *B* = 1.13, 95% CI: 0.57, 1.69; men widowed ≥ 20 years: *B* = 1.16, 95% CI: 0.36, 1.97). For women living in multigenerational households, widowhood status/duration was not associated with depression. Among women living in non-multigenerational households, those who were widowed within 0–9 years had higher odds of depression compared to those who are currently married (*B* = 1.99, 95% CI: 1.34, 2.97). The associations between widowhood status/duration and worse cognitive function were not statistically significant among men and women living in non-multigenerational households. All widowhood durations except mid-term widows of 10–19 years, were associated with worse cognitive function among men and women living in multigenerational households compared to their peers who were currently married.

Figure S2, S3, S4 and S5 present the interactions between widowhood status/duration and rural-urban and multigenerational living on depression and worse cognitive function among men and women. The risk of depression was more pronounced among women widowed for 0–9 years living in non-multigenerational households compared to their peers living in multigenerational households. Additionally, associations between widowhood status/duration and worse cognitive function were more pronounced among men and women residing in rural areas as well as among women living in non-multigenerational households.

## Discussion

This study examined the associations between widowhood status/duration, depression and cognitive function among older Indian adults age ≥ 60 years. The study also examined sex differences and differences by residential factors (i.e., rural/urban residence and multigenerational household status) in these associations. We found that recent widowhood was associated with higher levels of depression and worse cognitive functioning. While the negative effects on cognition diminished over time for men, it persisted for women. These effects were more pronounced in non-multigenerational households, with mixed patterns across urban and rural settings.

Marital union is documented as a social integrating factor that provides socioeconomic and psychological benefits and promotes mental well-being [62]. Previous studies have shown that spousal death may cause loneliness, resulting in increased depressive symptoms [63]. Similarly, we found that being widowed was significantly associated with higher odds of depression among men and women aged ≥ 60 years, consistent with previous findings [64]. In addition, a previous study found that being married provides daily cognitive stimulation that is conducive to maintaining better cognitive functioning among middle-aged and older married individuals [18]. Our finding that widowhood is significantly associated with worse cognitive function is consistent with studies that found that widowed Asian individuals had higher rates of cognitive impairment, Alzheimer’s disease, and dementia than their married counterparts [65–67].

Duration of widowhood on the other hand becomes important while examining the linkage between widowhood and mental health outcomes [68]. Evidence suggests that depression is strongly associated with the first few months or years of spousal loss [69, 70]. Several cross-sectional and longitudinal studies report women being more depressed due to widowhood [68, 71–73]. Consistent with the crisis model of distress [30, 31], our findings indicate that widowed individuals, particularly those with 0–9 years of bereavement, face an elevated risk of depression and those women with 20 + years of bereavement have worse cognitive function, supporting the model’s premise that acute psychological distress following spousal loss accelerates cognitive deterioration. The association between widowhood duration and mental and cognitive health outcomes in the present study is also in agreement with multiple longitudinal studies that found that compared to married individuals, those who were widowed for less than two years (recent widows) were more likely to have depression, and those who were widowed for more than five years were more likely to have significant cognitive deficits [69, 74].

Furthermore, we found that the link between widowhood duration (for 20 + years) and worse cognitive performance was more pronounced among older women with depression than among those without. The mechanisms linking widowhood duration and cognitive impairment may differ, depending on whether individuals experience depression. Psychological distress and loneliness following spousal loss can contribute to cognitive decline by triggering prolonged stress and neurobiological dysregulation [75–77]. Chronic stress activates the hypothalamic-pituitary-adrenal axis (HPA), leading to cortisol release, which can damage the hippocampus, impair memory, and accelerate cognitive decline, particularly in widowed individuals who develop depression [78, 79]. Depression is most intense in the initial years of widowhood [69, 70], contributing to cognitive deterioration through both behavioral and neurobiological mechanisms. Factors such as reduced social engagement, withdrawal from mentally stimulating activities, and decreased motivation may further exacerbate cognitive decline [80, 81]. However, widowed individuals who do not experience depression may still undergo gradual cognitive decline because of reduced mental stimulation, disengagement from cognitively enriching activities, and decreased social interactions that were previously sustained in marriage [82–85]. These findings highlight the significance of widowhood duration on cognitive health.

Our findings on the differences by rural-urban residence were mixed. Older men and women from both rural and urban areas experienced depression. Older men who were widowed for 0–9 years in rural areas and those widowed for 20 + years in urban areas showed worse cognitive function, while older women in both rural and urban areas exhibited poor cognitive function across most widowhood duration groups. These findings suggest that the duration of widowhood may have distinct impacts on cognitive health for men in rural and urban settings, and women across both settings appear to experience prolonged cognitive decline. However, such relationships in findings from Western studies differ due to different sociocultural contexts, in which roles and responsibilities do not have a positive impact on older women [73, 86, 87].

On the other hand, co-residence with adult children could alleviate mental distress related to spousal loss and lead to better cognitive scores, as was found among Korean women [88]. In agreement with this, our findings showed that living with children (multigenerational living) was a protective factor against depression among older women (widowed 0–9 years). Evidence suggests that women generally draw social support from diverse sources, including friends, relatives, and children [89, 90], as well as from their spiritual grounding, which may bring a strong sense of purpose, provide strength to live, and stay happy after widowhood [91, 92]. Furthermore, lack of access to social networks and social support are associated with poor cognitive function in later life [20, 93, 94]. However, contrary to this, we found significant association between widowhood and worse cognitive functioning among men and women in multigenerational households than their peers in non-multigenerational households which needs to be further investigated.

We acknowledge a few limitations to our study. First, given the cross-sectional nature of this study, we cannot infer causality from any associations. Second, the analysis did not consider unmarried partners, information on earlier marriages, quality of marital relationships, cause of spousal death, or caregiver status, all of which are known to influence depressive and cognitive outcomes [95–97]. Third, we were unable to adjust for pre-widowhood mental health status, which is likely a significant factor in determining health outcomes following spousal deaths.

Nevertheless, our study has several strengths. Considering previous research conducted by Jain et al. [25] and Srivastava et al. [45], which utilized the same dataset, our study contributes new knowledge on gender differences in widowhood experiences and explores differences in residential factors, that is, urban/rural residence and multigenerational households. The dataset for this study was obtained from a large, nationally representative, ethnically diverse, population-based comprehensive survey with a detailed assessment of depression and cognitive function. The richness of this information also allowed us to obtain a thorough understanding of the various aspects associated with mental and cognitive health outcomes among older Indian men and women, as a function of their marital relationships.

## Conclusions

Our findings highlight that older Indians, particularly women, are disadvantaged with respect to widowhood status/duration, which is associated with worse mental and cognitive health. Specifically, men and women who struggle to adjust to such transitions may be particularly at risk of adverse mental and cognitive health outcomes, more visible in terms of depression in the short term and cognitive impairment in the long term post-widowhood. Our findings also suggest that the associations between widowhood status/duration, depression, and worse cognitive function are stronger among men and women living in non-multigenerational households. Moderations by urban/rural residence were mixed. Incorporating information on marital status and transitions into the design of intervention programs for the aging population may help to better target potential beneficiaries. The findings may also have broader implications for other countries worldwide in terms of identifying and targeting subpopulations who are vulnerable to depressive symptoms and cognitive impairment post-bereavement. Further, future research on widowhood-associated depression and cognitive impairment should focus on specific subgroups that may be at greater risk, such as those without children and living in rural areas, with a focus on gendered pathways to widowhood.

## Electronic supplementary material

Below is the link to the electronic supplementary material.

Supplementary Material 1

## Data Availability

The data are available in the public domain and freely accessible from the Gateway to Global Aging Data (www.g2aging.org). The data are also available in the International Institute for Population Sciences (IIPS) data repository of Longitudinal Aging Study in India, upon request to IIPS, Mumbai, https://www.iipsindia.ac.in/content/LASI-data.

## Abbreviations

ADL: Activities of daily living
IADL: Instrumental activities of daily living
MPCE: Monthly per Capita Expenditure
AOR: Adjusted Odds Ratio
LASI: Longitudinal Aging Study in India
CI: Confidence Interval

## References

[1] Hertog S, Gerland P, Wilmoth J (2023) *India overtakes China as the world’s most populous country*. https://www.un.org/development/desa/pd/sites/www.un.org.development.desa.pd/files/undesa_pd_2023_policy-brief-153.pdf

[2] Palloni A (2001) Living arrangements of older persons. United Nations Population Bulletin

[3] Verbrugge LM (1985) Gender and health: An update on hypotheses and evidence. In *Journal of health and social behavior*. 10.2307/2136750 3905939

[4] Kashyap R, Behrman J (2020) Gender discrimination and excess female Under-5 mortality in india: A new perspective using Mixed-Sex twins. Demography 57(6):2143–2167. 10.1007/s13524-020-00909-0 32978723 10.1007/s13524-020-00909-0 PMC7732804

[5] Pal A, Yadav J, Kumari D, Jitenkumar Singh K (2020) Gender differentials and risk of infant and under five mortality in india. A comparative survival analysis. Child Youth Serv Rev 118:105477. 10.1016/j.childyouth.2020.105477

[6] Carr D, House JS, Kessler RC, Nesse RM, Sonnega J, Wortman C (2000) Marital quality and psychological adjustment to widowhood among older adults: A longitudinal analysis. Journals Gerontol Ser B: Psychol Sci Social Sci 55(4):S197–S207 10.1093/geronb/55.4.s197 11584882

[7] Holmes TH, Rahe RH (1967) The social readjustment rating scale. Journal of Psychosom atic Research, 11(2), 213-218. https://psycnet.apa.org/record/1968-03998-001 10.1016/0022-3999(67)90010-4 6059863

[8] Carr D (2004) Gender, Preloss Marital Dependence, and Older Adults’ Adjustment to Widowhood. In *Journal of Marriage and Family*. 10.1111/j.0022-2445.2004.00016.x

[9] Chen M, Dreze J (1992) Widows and Health in Rural North India. *Economic and Political Weekly*

[10] Jensen RT (2005) Caste, culture, and the status and well-being of widows in India. University of Chicago Press Chicago

[11] Johnson PS, Johnson JA (2001) The oppression of women in India. Violence against Woman. 10.1177/10778010122182893

[12] Carr D, Bodnar-Deren S (2009) Gender, Aging and Widowhood. In *International Handbook of Population Aging*. 10.1007/978-1-4020-8356-3_32

[13] Choi NG, Bohman TM (2007) Predicting the changes in depressive symptomatology in later life: how much do changes in health status, marital and caregiving status, work and volunteering, and health-related behaviors contribute? J Aging Health. 10.1177/0898264306297602 17215206 10.1177/0898264306297602

[14] Lee GR, DeMaris A, Bavin S, Sullivan R (2001) Gender differences in the depressive effect of widowhood in later life. *Journals of Gerontology - Series B Psychological Sciences and Social Sciences*. 10.1093/geronb/56.1.S56 10.1093/geronb/56.1.s56 11192346

[15] Lichtenstein P, Gatz M, Pedersen NL, Berg S, McClearn GE (1996) A co-twin-control study of response to widowhood. *Journals of Gerontology - Series B Psychological Sciences and Social Sciences*. 10.1093/geronb/51b.5.p279 10.1093/geronb/51b.5.p279 8809004

[16] Lee GR, DeMaris A (2007) Widowhood, gender, and depression. Res Aging. 10.1177/0164027506294098

[17] Feng L, Ng X-T, Yap P, Li J, Lee T-S, Håkansson K, Kua E-H, Ng T-P (2014) Marital status and cognitive impairment among Community-Dwelling Chinese older adults: the role of gender and social engagement. Dement Geriatric Cogn Disorders Extra 4(3):375–384. 10.1159/000358584 10.1159/000358584 PMC4241637 25473404

[18] Mousavi-Nasab SMH, Kormi-Nouri R, Sundström A, Nilsson LG (2012) The effects of marital status on episodic and semantic memory in healthy middle-aged and old individuals. Scand J Psychol 53(1):1–8. 10.1111/j.1467-9450.2011.00926.x 22092006 10.1111/j.1467-9450.2011.00926.x

[19] Rosnick CB, Small BJ, Burton AM (2010) The effect of spousal bereavement on cognitive functioning in a sample of older adults. Aging Neuropsychol Cognition 17(3):257–269. 10.1080/13825580903042692 10.1080/13825580903042692 19634026

[20] Zhang Z, Li LW, Xu H, Liu J (2019) Does widowhood affect cognitive function among Chinese older adults? SSM - Popul Health. 10.1016/j.ssmph.2018.100329 30581964 10.1016/j.ssmph.2018.100329 PMC6293047

[21] Chandrasekhar CP, Ghosh J (2017) Widowhood in India. *Business Line*

[22] Perkins JM, Lee H, James KS, Oh J, Krishna A, Heo J, Lee J, Subramanian SV (2016) Marital status, widowhood duration, gender and health outcomes: A cross-sectional study among older adults in India. BMC Public Health 16(1):1032. 10.1186/s12889-016-3682-9 27716203 10.1186/s12889-016-3682-9 PMC5045657

[23] Sreerupa, Rajan SI (2010) Gender and widowhood: disparity in health status and health care utilization among the aged in India. J Ethnic Cult Divers Social Work 19(4):287–304

[24] Sahoo DM (2014) An analysis of widowhood in india: A global perspective. Int J Multidisciplinary Curr Res. 2(3), 45-58

[25] Jain U, Liu H, Langa KM, Farron M, Kabeto M, Lee J (2022) Widowhood and cognition among older women in india: new insights on widowhood duration and mediators. SSM - Popul Health 19:101242. 10.1016/j.ssmph.2022.101242 36193099 10.1016/j.ssmph.2022.101242 PMC9525895

[26] Samanta T, Chen F, Vanneman R (2015) Living arrangements and health of older adults in India. Journals Gerontology: Ser B 70(6):937–947. 10.1093/geronb/gbu164 10.1093/geronb/gbu164 25452403

[27] Aroonsrimorakot S, Laiphrakpam M, Metadilogkul O, Konjengbam S (2019) Ageing, social isolation, loneliness, health, social care and longevity: insights from case studies in Thailand and India. Ageing Int 44(4):371–384. 10.1007/s12126-019-09353-x

[28] Chokkanathan S (2024) Family environment, loneliness, hope, and subjective Well-Being of Asian older adults. Int J Aging Hum Dev 98(2):208–220. 10.1177/00914150231171839 37122151 10.1177/00914150231171839

[29] Sekher TV (2012) Rural Demography of India. In L. J. Kulcsár & K. J. Curtis (Eds.), *International Handbook of Rural Demography* (Vol. 3, pp. 169–189). Springer Netherlands. 10.1007/978-94-007-1842-5_13

[30] Halpern HA (1973) Crisis theory: A definitional study. Commun Ment Health J 9(4):342–349. 10.1007/BF01410870 10.1007/BF01410870 4148620

[31] Kim Y, Kim C-S (2016) Will the pain of losing a husband last forever? The effect of transition to widowhood on mental health. Dev Soc 45(1):165–187

[32] Neimeyer RA (2006) Widowhood, grief and the quest for meaning. Spousal Bereave Late Life, 6:227–252

[33] International Institute for Population Sciences (IIPS), MoHFW NPHCE, Harvard TH (2020) Chan School of Public Health (HSPH), & The university of Southern California (USC). Longitudinal Ageing Study in India (LASI) Wave 1. In *India Report*

[34] Kessler RC, Andrews G, Mroczek D, Ustun B, Wittchen H (1998) (CIDI‐SF). Int J Methods Psychiatr Res 7(4):171–185. 10.1002/mpr.47. The World Health Organization Composite International Diagnostic Interview short-form 10.1002/mpr.168 PMC6878592 15297906

[35] Dang L, Dong L, Mezuk B (2020) Shades of blue and gray: A comparison of the center for epidemiologic studies depression scale and the composite international diagnostic interview for assessment of depression syndrome in later life. Gerontologist 60(4):e242–e253. 10.1093/geront/gnz044 31112598 10.1093/geront/gnz044 PMC7228460

[36] Kessler RC, Bromet EJ (2013) The epidemiology of depression across cultures. Annu Rev Public Health 34:119–138. 10.1146/annurev-publhealth-031912-114409 23514317 10.1146/annurev-publhealth-031912-114409 PMC4100461

[37] Trainor K, Mallett J, Rushe T (2013) Age related differences in mental health scale scores and depression diagnosis: adult responses to the CIDI-SF and MHI-5. J Affect Disord 151(2):639–645. 10.1016/j.jad.2013.07.011 23993442 10.1016/j.jad.2013.07.011

[38] Juva K, MÄKELÄ M, Erkinjuntti T, Sulkava R, Valvanne YUKOSKIR, J., Tilvis R (1997) Functional assessment scales in detecting dementia. Age Ageing 26(5):393–400 9351484 10.1093/ageing/26.5.393

[39] Blankson AN, McArdle JJ (2014) A brief report on the factor structure of the cognitive measures in the HRS/AHEAD studies. *Journal of Aging Research*, *2014* 10.1155/2014/798514 PMC4058144 24971176

[40] Saenz JL, Adar SD, Zhang YS, Wilkens J, Chattopadhyay A, Lee J, Wong R (2021) Household use of polluting cooking fuels and late-life cognitive function: A harmonized analysis of india, mexico, and China. Environ Int 156:106722. 10.1016/j.envint.2021.106722 34182193 10.1016/j.envint.2021.106722 PMC8380666

[41] Gupta M, Gupta V, Buckshee N, R., Sharma V (2019) Validity and reliability of Hindi translated version of Montreal cognitive assessment in older adults. Asian J Psychiatry 45:125–128. 10.1016/j.ajp.2019.09.022 10.1016/j.ajp.2019.09.022 31586818

[42] Herzog AR, Wallace RB (1997) Measures of cognitive functioning in the AHEAD study. Journals Gerontology: Ser B 52B(SpecialIssue):37–48. 10.1093/geronb/52B.Special_Issue.37 10.1093/geronb/52b.special_issue.37 9215356

[43] Meng Q, Wang H, Strauss J, Langa KM, Chen X, Wang M, Qu Q, Chen W, Kuang W, Zhang N (2019) Validation of neuropsychological tests for the China health and retirement longitudinal study harmonized cognitive assessment protocol. Int Psychogeriatr 31(12):1709–1719 31309907 10.1017/S1041610219000693 PMC8082093

[44] Singham T, Bell G, Saunders R, Stott J (2021) Widowhood and cognitive decline in adults aged 50 and over: A systematic review and meta-analysis. Ageing Res Rev 71:101461 34534681 10.1016/j.arr.2021.101461

[45] Srivastava S, Debnath P, Shri N, Muhammad T (2021) The association of widowhood and living alone with depression among older adults in India. Sci Rep 11(1):21641 34737402 10.1038/s41598-021-01238-x PMC8568934

[46] Diener E, Emmons RA, Larsen RJ, Griffin S (1985) The satisfaction with life scale. J Pers Assess 49(1):71–75. 10.1207/s15327752jpa4901_13 16367493 10.1207/s15327752jpa4901_13

[47] Diener E, Inglehart R, Tay L (2013) Theory and validity of life satisfaction scales. Soc Indic Res 112(3):497–527. 10.1007/s11205-012-0076-y

[48] Cullati S, Mukhopadhyay S, Sieber S, Chakraborty A, Burton-Jeangros C (2018) Is the single self-rated health item reliable in india?? A construct validity study. BMJ Global Health, 3(6), p.e000856 10.1136/bmjgh-2018-000856 PMC6231101 30483411

[49] Cullati S, Bochatay N, Rossier C, Guessous I, Burton-Jeangros C, Courvoisier DS (2020) Does the single-item self-rated health measure the same thing across different wordings? Construct validity study. Qual Life Res 29(9):2593–2604. 10.1007/s11136-020-02533-2 32436111 10.1007/s11136-020-02533-2 PMC7434800

[50] Manor O, Matthews S, Power C (2000) Dichotomous or categorical response? Analysing self-rated health and lifetime social class. Int J Epidemiol 29(1):149–157 10750617 10.1093/ije/29.1.149

[51] Power C, Matthews S, Manor O (1996) Inequalities in self rated health in the 1958 birth cohort: lifetime social circumstances or social mobility? BMJ 313(7055):449–453 8776310 10.1136/bmj.313.7055.449 PMC2351851

[52] Pashmdarfard M, Azad A (2020) Assessment tools to evaluate activities of daily living (ADL) and instrumental activities of daily living (IADL) in older adults: A systematic review. Med J Islamic Repub Iran 34:33. 10.34171/mjiri.34.33 10.34171/mjiri.34.33 PMC7320974 32617272

[53] Muhammad T, Srivastava S, Muneera K, Kumar M, Kelekar U (2024) Treatment for insomnia symptoms is associated with reduced depression among older adults: A propensity score matching approach. Clin Gerontologist 47(3):436–451. 10.1080/07317115.2023.2208582 10.1080/07317115.2023.2208582 37153958

[54] Wardle-Pinkston S, Slavish DC, Taylor DJ (2019) Insomnia and cognitive performance: A systematic review and meta-analysis. Sleep Med Rev 48:101205 31522135 10.1016/j.smrv.2019.07.008

[55] Ding B, Small M, Bergström G, Holmgren U (2017) A cross-sectional survey of night-time symptoms and impact of sleep disturbance on symptoms and health status in patients with COPD. Int J Chronic Obstr Pulm Dis 12:589–599. 10.2147/COPD.S122485 10.2147/COPD.S122485 PMC5315208 28243077

[56] Lallukka T, Sares-Jäske L, Kronholm E, Sääksjärvi K, Lundqvist A, Partonen T, Rahkonen O, Knekt P (2012) Sociodemographic and socioeconomic differences in sleep duration and insomnia-related symptoms in Finnish adults. BMC Public Health 12(1):565. 10.1186/1471-2458-12-565 22839359 10.1186/1471-2458-12-565 PMC3490788

[57] Gupta D (2000) Interrogating caste: Understanding hierarchy and difference in Indian society. Penguin Books India

[58] Jodhka SS (2016) Ascriptive hierarchies: caste and its reproduction in contemporary India. Curr Sociol 64(2):228–243. 10.1177/0011392115614784

[59] Hopkins EW (2020) The religions of India. BoD– Books on Demand

[60] Pew Research Center (2021) *Measuring caste in India*. https://www.pewresearch.org/decoded/2021/06/29/measuring-caste-in-india/

[61] Zacharias A, Vakulabharanam V (2011) Caste stratification and wealth inequality in India. World Dev. 10.1016/j.worlddev.2011.04.026

[62] Ertel KA, Glymour MM, Berkman LF (2008) Effects of social integration on preserving memory function in a nationally representative US elderly population. Am J Public Health 98(7):1215–1220. 10.2105/AJPH.2007.113654 18511736 10.2105/AJPH.2007.113654 PMC2424091

[63] Fried EI, Bockting C, Arjadi R, Borsboom D, Amshoff M, Cramer AOJ, Epskamp S, Tuerlinckx F, Carr D, Stroebe M (2015) From loss to loneliness: the relationship between bereavement and depressive symptoms. J Abnorm Psychol 124(2):256–265. 10.1037/abn0000028 25730514 10.1037/abn0000028

[64] Giri M, Chen T, Yu W, Lü Y (2016) Prevalence and correlates of cognitive impairment and depression among elderly people in the world’s fastest growing city, chongqing, people’s Republic of China. Clin Interv Aging 11:1091–1098. 10.2147/CIA.S113668 27574409 10.2147/CIA.S113668 PMC4990376

[65] Bae J, Bin, Kim YJ, Han JW, Kim TH, Park JH, Lee SB, Lee JJ, Jeong HG, Kim JL, Jhoo JH, Yoon JC, Kim KW (2015) Incidence of and risk factors for alzheimer’s disease and mild cognitive impairment in Korean elderly. Dement Geriatr Cogn Disord. 10.1159/000366555 25401488 10.1159/000366555

[66] Fan LY, Sun Y, Lee HJ, Yang SC, Chen TF, Lin KN, Lin CC, Wang PN, Tang LY, Chiu MJ (2015) Marital status, lifestyle and dementia: A nationwide survey in Taiwan. PLoS ONE 10(9):1–11. 10.1371/journal.pone.0139154 10.1371/journal.pone.0139154 PMC4587383 26413719

[67] Zhang ZX, Zahner GEP, Román GC, Liu XH, Wu CB, Hong Z, Hong X, Tang MN, Zhou B, Qu QM, Zhang XJ, Li H (2006) Socio-demographic variation of dementia subtypes in china: methodology and results of a prevalence study in beijing, chengdu, shanghai, and Xian. Neuroepidemiology. 10.1159/000096131 17035714 10.1159/000096131

[68] Perrig-Chiello P, Spahni S, Höpflinger F, Carr D (2016) Cohort and gender differences in psychosocial adjustment to later-life widowhood. Journals Gerontol - Ser B Psychol Sci Social Sci 71(4):765–774. 10.1093/geronb/gbv004 10.1093/geronb/gbv004 25731182

[69] Vable AM, Subramanian SV, Rist PM, Glymour MM (2016) Does the widowhood effect precede spousal bereavement? Results from a nationally representative sample of older adults. Physiol Behav 176(1):100–106. 10.1016/j.jagp.2014.05.004.Does 10.1016/j.jagp.2014.05.004 PMC5511695 24974142

[70] Zisook S, Shuchter SR (1993) Major depression associated with widowhood. Am J Geriatric Psychiatry 1(4):316–326. 10.1097/00019442-199300140-00006 10.1097/00019442-199300140-00006 28530910

[71] Burns RA, Browning CJ, Kendig HL (2015) Examining the 16-year trajectories of mental health and wellbeing through the transition into widowhood. Int Psychogeriatr 27(12):1979–1986. 10.1017/S1041610215000472 25851736 10.1017/S1041610215000472

[72] Jadhav A, Weir D (2018) Widowhood and depression in a Cross-National perspective: evidence from the united states, europe, korea, and China. Journals Gerontol - Ser B Psychol Sci Social Sci 73(8):e143–e153. 10.1093/geronb/gbx021 10.1093/geronb/gbx021 PMC6178968 28329854

[73] Vidarsdottir H, Fang F, Chang M, Aspelund T, Fall K, Jonsdottir MK, Jonsson PV, Cotch MF, Harris TB, Launer LJ, Gudnason V, Valdimarsdottir U (2014) Spousal loss and cognitive function in later life: A 25-year follow-up in the AGES-Reykjavik study. Am J Epidemiol 179(6):674–683. 10.1093/aje/kwt321 24444551 10.1093/aje/kwt321 PMC3939848

[74] Shin SH, Kim G, Park S (2018) Widowhood status as a risk factor for cognitive decline among older adults. Am J Geriatric Psychiatry 26(7):778–787. 10.1016/j.jagp.2018.03.013 10.1016/j.jagp.2018.03.013 29748078

[75] Harrington KD, Vasan S, Kang JE, Sliwinski MJ, Lim MH (2023) Loneliness and cognitive function in older adults without dementia: A systematic review and Meta-Analysis. J Alzheimer’s Disease 91(4):1243–1259. 10.3233/JAD-220832 36617781 10.3233/JAD-220832 PMC9983432

[76] Kwon D-Y, Jung J-M, Park MH (2017) Loneliness in elderly patients with mild cognitive impairment: A pilot study. Dement Neurocognitive Disorders 16(4):110–113 10.12779/dnd.2017.16.4.110 PMC6428005 30906381

[77] Zhong B-L, Chen S-L, Conwell Y (2016) Effects of transient versus chronic loneliness on cognitive function in older adults: findings from the Chinese longitudinal healthy longevity survey. Am J Geriatric Psychiatry 24(5):389–398 10.1016/j.jagp.2015.12.009 PMC4846538 26905049

[78] Monk TH, Pfoff MK, Zarotney JR (2013) Depression in the Spousally Bereaved Elderly: Correlations with Subjective Sleep Measures. *Depression Research and Treatment*, *2013*, 1–4. 10.1155/2013/409538 10.1155/2013/409538 PMC3619539 23634298

[79] Zheng F, Zhong B, Song X, Xie W (2018) Persistent depressive symptoms and cognitive decline in older adults. Br J Psychiatry 213(5):638–644 30132434 10.1192/bjp.2018.155

[80] Bi T, Kou H, Kong Y, Shao B (2022) Widowhood impairs emotional cognition among elderly. Front Aging Neurosci 13:808885 35173602 10.3389/fnagi.2021.808885 PMC8841410

[81] Mingyuan S, Young K, Li Y, Yeyuan Z, Wang J, Shuhan J (2024) The influence of widowhood and social engagement on cognitive impairment among Chinese older adults and factors mediating their association. *Journal of Global Health*, *14*. https://search.proquest.com/openview/e9cd3acad5d0a8bd14ef190d46cc5435/1?pq-origsite=gscholar%26cbl=2045580%26casa_token=PL10jr-9nU0AAAAA:qAbU0PTQa-5H-mxXLwAZ_fH-W16yg0kudZwMeDKPtka60GGeIUpa7ZNrOieyM9k1nzg1EN82 10.7189/jogh.14.04193 PMC11413616 39301589

[82] Hsiao Y-H, Lee M-C, Yeh C-J, Tai C-J, Lee S-S (2021) Social participation and survival in widowed persons: results of the Taiwan longitudinal study on aging. Int J Environ Res Public Health 18(20):10974 34682721 10.3390/ijerph182010974 PMC8535271

[83] Kim YB, Lee SH (2019) Social network types and cognitive decline among older Korean adults: A longitudinal population-based study. Int J Geriatr Psychiatry 34(12):1845–1854. 10.1002/gps.5200 31418470 10.1002/gps.5200

[84] Shen S, Cheng J, Li J, Xie Y, Wang L, Zhou X, Zhou W, Zhu L, Wang T, Tu J, Bao H, Cheng X (2022) Association of marital status with cognitive function in Chinese hypertensive patients: A cross-sectional study. BMC Psychiatry 22(1):504. 10.1186/s12888-022-04159-9 35897015 10.1186/s12888-022-04159-9 PMC9327272

[85] Xiang N, Liu E, Li H, Qin X, Liang H, Yue Z (2021) The association between widowhood and cognitive function among Chinese elderly people: Do gender and widowhood duration make a difference? *Healthcare*, *9*(8), 991. https://www.mdpi.com/2227-9032/9/8/991 10.3390/healthcare9080991 PMC8392527 34442128

[86] Karlamangla AS, Miller-Martinez D, Aneshensel CS, Seeman TE, Wight RG, Chodosh J (2009) Trajectories of cognitive function in late life in the united states: demographic and socioeconomic predictors. Am J Epidemiol 170(3):331–342. 10.1093/aje/kwp154 19605514 10.1093/aje/kwp154 PMC2727175

[87] Sommerlad A, Ruegger J, Singh-Manoux A, Lewis G, Livingston G (2018) Marriage and risk of dementia: systematic review and meta-analysis of observational studies. J Neurol Neurosurg Psychiatry 89(3):231–238. 10.1136/jnnp-2017-316274 29183957 10.1136/jnnp-2017-316274 PMC5869449

[88] Do YK, Malhotra C (2012) The effect of coresidence with an adult child on depressive symptoms among older widowed women in South Korea: An instrumental variables estimation. *Journals of Gerontology - Series B Psychological Sciences and Social Sciences*, *67 B*(3), 384–391. 10.1093/geronb/gbs033 10.1093/geronb/gbs033 22421809

[89] Caetano SC, Silva CM, Vettore MV (2013) Gender differences in the association of perceived social support and social network with self-rated health status among older adults: A population-based study in Brazil. BMC Geriatr 13(1). 10.1186/1471-2318-13-122 10.1186/1471-2318-13-122 PMC4225700 24229389

[90] Pillemer S, Ayers E, Holtzer R (2019) Gender-stratified analyses reveal longitudinal associations between social support and cognitive decline in older men. Aging Ment Health 23(10):1326–1332 30328696 10.1080/13607863.2018.1495178 PMC6470062

[91] Corsentino EA, Collins N, Sachs-Ericsson N, Blazer DG (2009) Religious attendance reduces cognitive decline among older women with high levels of depressive symptoms. Journals Gerontol - Ser Biol Sci Med Sci 64(12):1283–1289. 10.1093/gerona/glp116 10.1093/gerona/glp116 PMC2773810 19675176

[92] Stanley MA, Bush AL, Camp ME, Jameson JP, Phillips LL, Barber CR, Zeno D, Lomax JW, Cully JA (2011) Older adults’ preferences for religion/spirituality in treatment for anxiety and depression. Aging Ment Health 15(3):334–343 21491218 10.1080/13607863.2010.519326

[93] Ellwardt L, Aartsen M, Deeg D, Steverink N (2013) Does loneliness mediate the relation between social support and cognitive functioning in later life? Soc Sci Med 98:116–124. 10.1016/j.socscimed.2013.09.002 24331889 10.1016/j.socscimed.2013.09.002

[94] Gow AJ, Corley J, Starr JM, Deary IJ (2013) Which social network or support factors are associated with cognitive abilities in old age? Gerontology 59(5):454–463. 10.1159/000351265 23711796 10.1159/000351265

[95] Haghighi P, Littler EA, Mauer-Vakil D, Miller M, Oremus M (2024) Exploring the relationship between marital quality and cognitive function: A systematic review. Soc Sci Med, 117120 10.1016/j.socscimed.2024.117120 39019001

[96] Schaan B (2013) Widowhood and depression among older Europeans—The role of gender, caregiving, marital quality, and regional context. Journals Gerontol Ser B: Psychol Sci Social Sci 68(3):431–442 10.1093/geronb/gbt015 PMC3627659 23591571

[97] Wu-Chung EL, Leal SL, Denny BT, Cheng SL, Fagundes CP (2022) Spousal caregiving, widowhood, and cognition: A systematic review and a biopsychosocial framework for Understanding the relationship between interpersonal losses and dementia risk in older adulthood. Neurosci Biobehavioral Reviews 134:104487 10.1016/j.neubiorev.2021.12.010 PMC8925984 34971701

