# Supplementary material for: Associations between widowhood status/duration, depression, and cognitive function among community-dwelling Indians age 60 years or older: Exploration of sex and residential factors
Source: Soc Psychiatry Psychiatr Epidemiol. 2025 Jun 27;60(10):2401–17. doi: 10.1007/s00127-025-02950-z (PMC12449418; doi:10.1007/s00127-025-02950-z)
Supplement: Supplementary file 1 — Supplementary Material 1 [file 127_2025_2950_MOESM1_ESM.docx]

**Supplemental materials**

| **Table-S1** Multivariate logistic and linear regression estimates of depression and worse cognitive function by widowhood status/duration (5-year groups) and other background characteristics among older men and women in India, LASI, 2017-2019 | | | | | | | | |  |
| --- | --- | --- | --- | --- | --- | --- | --- | --- | --- |
| **Background characteristic** | | **Men** | | | **Women** | | | |  |
|  |  | **Depression, AOR (95%CI)** | **Worse cognitive function, b (95%CI)** | | **Depression, AOR (95%CI)** | | **Worse cognitive function, b (95%CI)** | |  |
| **Widowhood status/duration** | |  |  | |  | |  | |  |
| Currently married | | Ref. | Ref. | | Ref. | | Ref. | |  |
| Widows (0-4 years) | | 2.47*** (1.74, 3.53) | 0.80** (0.20, 1.41) | | 1.92*** (1.47, 2.51) | | 0.56* (0.10, 1.01) | |  |
| Widows (5-9 years) | | 0.58 (0.30, 1.11) | 0.81* (0.03, 1.59) | | 1.22 (0.88, 1.68) | | 0.54* (0.09, 1.00) | |  |
| Widows (10-14 years) | | 1.16 (0.63, 2.13) | 0.01 (-0.83, 0.85) | | 1.25 (0.82, 1.91) | | 0.86* (0.15, 1.56) | |  |
| Widows (15-19 years) | | 0.50 (0.20, 1.28) | -0.18 (-1.17, 0.80) | | 1.00 (0.70, 1.43) | | 0.23 (-0.28, 0.75) | |  |
| Widows (20+ years) | | 0.66 (0.40, 1.10) | 0.67 (-0.40, 1.74) | | 1.09 (0.84, 1.42) | | 0.90*** (0.44, 1.36) | |  |
| **Age** | |  |  | |  | |  | |  |
| 60-69 years | | Ref. | Ref. | | Ref. | | Ref. | |  |
| 70-79 years | | 0.66*** (0.53, 0.82) | 0.99*** (0.65, 1.33) | | 0.94 (0.76, 1.16) | | 1.20*** (0.83, 1.56) | |  |
| > 80 years | | 0.69 (0.44, 1.09) | 1.91*** (1.29, 2.52) | | 0.98 (0.71, 1.36) | | 2.59*** (2.01, 3.16) | |  |
| **Education** | |  |  | |  | |  | |  |
| No formal education | | Ref. | Ref. | | Ref. | | Ref. | |  |
| Primary not completed | | 1.24 (0.87, 1.77) | -3.32*** (-3.80, -2.83) | | 1.22 (0.89, 1.69) | | -3.17*** (-3.64, -2.69) | |  |
| Primary | | 1.13 (0.86, 1.50) | -4.64*** (-5.08, -4.19) | | 1.30 (0.94, 1.79) | | -5.54*** (-6.11, -4.97) | |  |
| Secondary/higher | | 1.06 (0.81, 1.38) | -6.86*** (-7.24, -6.49) | | 1.07 (0.72, 1.58) | | -7.89*** (-8.52, -7.26) | |  |
| **Multigenerational living** | |  |  | |  | |  | |  |
| Yes | | Ref. | Ref. | | Ref. | | Ref. | |  |
| No | 1.03 (0.82, 1.29) | | | 0.23 (-0.11, 0.56) | | 1.29* (1.05, 1.56) | | 0.39* (0.07, 0.71) | |
| **Working status** | |  |  | |  | |  | |  |
| Currently working | | Ref. | Ref. | | Ref. | | Ref. | |  |
| Retired | | 0.97 (0.57, 1.67) | -0.39 (-1.17, 0.39) | | 1.49*** (1.19, 1.86) | | -0.29 (-0.63, 0.04) | |  |
| Never worked | | 0.92 (0.54, 1.57) | -0.47 (-1.25, 0.31) | | 1.80*** (1.37, 2.37) | | -0.80*** (-1.19, -0.41) | |  |
| Currently not working | | 0.97 (0.54, 1.75) | -1.13** (-1.94, -0.32) | | 2.00* (1.11, 3.61) | | -2.35*** (-3.24, -1.46) | |  |
| **Social participation** | |  |  | |  | |  | |  |
| No | | Ref. | Ref. | | Ref. | | Ref. | |  |
| Yes | | 0.74 (0.48, 1.15) | -0.56** (-0.95, -0.17) | | 1.10 (0.71, 1.70) | | -0.83** (-1.40, -0.27) | |  |
| **Life satisfaction** | |  |  | |  | |  | |  |
| Low | | Ref. | Ref. | | Ref. | | Ref. | |  |
| Medium | | 0.61*** (0.46, 0.81) | -0.32 (-0.72, 0.07) | | 0.54*** (0.43, 0.67) | | -0.73*** (-1.10, -0.36) | |  |
| High | | 0.43*** (0.34, 0.53) | -0.45* (-0.81, -0.10) | | 0.57*** (0.46, 0.72) | | -0.88*** (-1.20, -0.56) | |  |
| **Self-rated health** | |  |  | |  | |  | |  |
| Good | | Ref. | Ref. | | Ref. | | Ref. | |  |
| Poor | | 2.18*** (1.75, 2.72) | 0.86*** (0.50, 1.21) | | 2.12*** (1.74, 2.58) | | 0.53** (0.21, 0.86) | |  |
| **Difficulty with ADL** | |  |  | |  | |  | |  |
| No | | Ref. | Ref. | | Ref. | | Ref. | |  |
| Yes | | 1.71*** (1.28, 2.30) | 0.37 (-0.08, 0.82) | | 1.44*** (1.17, 1.77) | | 0.44* (0.06, 0.81) | |  |
| **Difficulty with IADL** | |  |  | |  | |  | |  |
| No | | Ref. | Ref. | | Ref. | | Ref. | |  |
| Yes | | 1.52** (1.14, 2.02) | 1.22*** (0.85, 1.59) | | 1.69*** (1.37, 2.09) | | 1.02*** (0.69, 1.34) | |  |
| **Insomnia symptoms** | |  |  | |  | |  | |  |
| No | | Ref. | Ref. | | Ref. | | Ref. | |  |
| Yes | | 1.89*** (1.54, 2.31) | 0.06 (-0.24, 0.37) | | 2.09*** (1.73, 2.52) | | 0.05 (-0.23, 0.34) | |  |
| **MPCE quintile** | |  |  | |  | |  | |  |
| Poorest | | Ref. | Ref. | | Ref. | | Ref. | |  |
| Poorer | | 0.81 (0.60, 1.10) | 0.13 (-0.29, 0.55) | | 1.02 (0.79, 1.32) | | -0.32 (-0.74, 0.10) | |  |
| Middle | | 1.21 (0.85, 1.73) | -0.31 (-0.75, 0.12) | | 0.93 (0.69, 1.25) | | -0.63** (-1.06, -0.21) | |  |
| Richer | | 1.27 (0.93, 1.75) | -0.29 (-0.75, 0.17) | | 1.11 (0.83, 1.49) | | -0.70** (-1.14, -0.25) | |  |
| Richest | | 1.47* (1.07, 2.02) | -0.56* (-1.03, -0.09) | | 1.29 (0.98, 1.70) | | -0.86** (-1.39, -0.32) | |  |
| **Religion** | |  |  | |  | |  | |  |
| Hindu | | Ref. | Ref. | | Ref. | | Ref. | |  |
| Muslim | | 0.93 (0.69, 1.26) | -0.32 (-0.82, 0.19) | | 1.10 (0.78, 1.54) | | 0.22 (-0.20, 0.65) | |  |
| Christian | | 0.64 (0.26, 1.59) | 0.56 (-0.23, 1.35) | | 2.14** (1.30, 3.53) | | -0.20 (-0.94, 0.54) | |  |
| Others | | 0.97 (0.61, 1.56) | 0.44 (-0.17, 1.06) | | 1.24 (0.80, 1.93) | | -0.58 (-1.18, 0.01) | |  |
| **Caste** | |  |  | |  | |  | |  |
| Scheduled Caste | | Ref. | Ref. | | Ref. | | Ref. | |  |
| Scheduled Tribe | | 0.52** (0.34, 0.80) | 1.17*** (0.53, 1.81) | | 0.55** (0.37, 0.81) | | 0.76** (0.21, 1.30) | |  |
| Other Backward Class | | 0.95 (0.72, 1.25) | -0.42* (-0.82, -0.01) | | 1.44** (1.12, 1.85) | | -0.58** (-0.98, -0.18) | |  |
| Others | | 0.86 (0.64, 1.15) | -0.25 (-0.68, 0.19) | | 1.14 (0.85, 1.54) | | -0.55* (-0.97, -0.12) | |  |
| **Place of residence** | |  |  | |  | |  | |  |
| Rural | | Ref. | Ref. | | Ref. | | Ref. | |  |
| Urban | | 0.98 (0.77, 1.25) | -1.19*** (-1.52, -0.85) | | 0.77* (0.60, 0.99) | | -1.44*** (-1.78, -1.10) | |  |
| **Region** | |  |  | |  | |  | |  |
| North | | Ref. | Ref. | | Ref. | | Ref. | |  |
| Central | | 1.92*** (1.43, 2.57) | -0.09 (-0.55, 0.37) | | 2.68*** (2.00, 3.57) | | -0.50* (-0.89, -0.11) | |  |
| East | | 0.96 (0.71, 1.31) | -0.11 (-0.50, 0.28) | | 1.04 (0.78, 1.37) | | -0.43* (-0.83, -0.03) | |  |
| Northeast | | 0.98 (0.58, 1.67) | -0.27 (-0.82, 0.28) | | 0.99 (0.66, 1.49) | | -0.40 (-0.94, 0.14) | |  |
| West | | 0.57** (0.40, 0.80) | -0.15 (-0.58, 0.29) | | 0.55*** (0.40, 0.77) | | -1.23*** (-1.70, -0.75) | |  |
| South | | 0.95 (0.65, 1.38) | 1.05*** (0.60, 1.51) | | 1.26 (0.92, 1.73) | | 0.98*** (0.54, 1.41) | |  |
| LASI, Longitudinal Aging Study in India; ADL: Activities of daily living; IADL: Instrumental activities of daily living; Ref: Reference; AOR: Adjusted Odds Ratio; * if p<0.05; CI: Confidence interval; MPCE: Monthly per capita consumption expenditure; Other Backward Classes refer to groups identified by the Indian government as socially and economically disadvantaged for the purpose of affirmative action policies. | | | | | | | | |  |

Figure S1: Interaction effect of widowhood status/duration (5-year groups) and depression on cognitive function among men and women

**Table-S2a** Multivariate logistic and linear regression estimates of depression and worse cognitive function by widowhood status/duration and other background characteristics among **men**, stratified by rural/urban place of residence, LASI, 2017-2019

| **Background characteristics** | Rural | | Urban | |
| --- | --- | --- | --- | --- |
|  | Depression | Worse cognitive function | Depression | Worse cognitive function |
| **Widowhood duration** |  |  |  |  |
| Currently married | Ref. | Ref. | Ref. | Ref. |
| Widows (0-9 years) | 1.59* (1.10, 2.31) | 0.82** (0.23, 1.41) | 1.89* (1.03, 3.45) | 0.84 (-0.02, 1.71) |
| Widows (10-19 years) | 0.77 (0.43, 1.39) | 0.09 (-0.61, 0.79) | 1.66 (0.62, 4.43) | -0.40 (-1.76, 0.97) |
| Widows (20+ years) | 0.56 (0.30, 1.04) | 0.50 (-0.79, 1.79) | 1.37 (0.60, 3.15) | 1.43* (0.24, 2.62) |
| **Age** |  |  |  |  |
| 60-69 years | Ref. | Ref. | Ref. | Ref. |
| 70-79 years | 0.65*** (0.50, 0.83) | 1.02*** (0.61, 1.42) | 0.62* (0.39, 0.98) | 0.79** (0.23, 1.35) |
| > 80 years | 0.69 (0.40, 1.17) | 1.73*** (0.97, 2.48) | 0.69 (0.35, 1.39) | 2.16*** (1.18, 3.14) |
| **Education** |  |  |  |  |
| No formal education | Ref. | Ref. | Ref. | Ref. |
| Primary not completed | 1.32 (0.89, 1.94) | -3.42*** (-3.97, -2.88) | 0.86 (0.46, 1.61) | -2.39*** (-3.31, -1.46) |
| Primary | 1.12 (0.82, 1.54) | -4.88*** (-5.35, -4.40) | 1.00 (0.54, 1.84) | -3.39*** (-4.37, -2.41) |
| Secondary/higher | 1.07 (0.79, 1.46) | -7.22*** (-7.65, -6.80) | 0.89 (0.54, 1.47) | -5.61*** (-6.38, -4.84) |
| **Multigenerational living** |  |  |  |  |
| Yes | Ref. | Ref. | Ref. | Ref. |
| No | 1.01 (0.79, 1.31) | 0.03 (-0.37, 0.43) | 1.21 (0.80, 1.83) | 0.74* (1.33, 0.16) |
| **Working status** |  |  |  |  |
| Currently working | Ref. | Ref. | Ref. | Ref. |
| Retired | 0.97 (0.52, 1.81) | -0.21 (-1.15, 0.72) | 0.67 (0.24, 1.86) | -0.91 (-2.23, 0.42) |
| Never worked | 0.91 (0.49, 1.68) | -0.36 (-1.30, 0.58) | 0.64 (0.22, 1.85) | -0.85 (-2.17, 0.47) |
| Currently not working | 1.13 (0.56, 2.27) | -1.18* (-2.19, -0.17) | 0.51 (0.18, 1.49) | -1.43* (-2.76, -0.10) |
| **Social participation** |  |  |  |  |
| No | Ref. | Ref. | Ref. | Ref. |
| Yes | 0.82 (0.48, 1.39) | -0.32 (-0.80, 0.16) | 0.62 (0.29, 1.29) | -0.94** (-1.59, -0.29) |
| **Life satisfaction** |  |  |  |  |
| Low | Ref. | Ref. | Ref. | Ref. |
| Medium | 0.59** (0.43, 0.83) | -0.33 (-0.79, 0.12) | 0.74 (0.45, 1.20) | -0.20 (-0.95, 0.55) |
| High | 0.41*** (0.32, 0.54) | -0.56** (-0.96, -0.16) | 0.55** (0.36, 0.86) | -0.07 (-0.77, 0.63) |
| **Self-rated health** |  |  |  |  |
| Good | Ref. | Ref. | Ref. | Ref. |
| Poor | 2.06*** (1.59, 2.67) | 0.81*** (0.40, 1.21) | 2.70*** (1.79, 4.07) | 1.19*** (0.52, 1.87) |
| **Difficulty with ADL** |  |  |  |  |
| No | Ref. | Ref. | Ref. | Ref. |
| Yes | 1.66** (1.16, 2.36) | 0.33 (-0.21, 0.86) | 1.89** (1.17, 3.07) | 0.42 (-0.31, 1.15) |
| **Difficulty with IADL** |  |  |  |  |
| No | Ref. | Ref. | Ref. | Ref. |
| Yes | 1.43* (1.03, 1.99) | 1.33*** (0.91, 1.75) | 2.00** (1.22, 3.28) | 0.84* (0.16, 1.51) |
| **Insomnia symptoms** |  |  |  |  |
| No | Ref. | Ref. | Ref. | Ref. |
| Yes | 1.87*** (1.48, 2.35) | -0.04 (-0.39, 0.31) | 1.84** (1.25, 2.69) | 0.41 (-0.12, 0.94) |
| **MPCE quintile** |  |  |  |  |
| Poorest | Ref. | Ref. | Ref. | Ref. |
| Poorer | 0.73 (0.51, 1.03) | -0.07 (-0.59, 0.44) | 1.12 (0.65, 1.95) | 0.58 (-0.09, 1.26) |
| Middle | 1.09 (0.72, 1.64) | -0.39 (-0.91, 0.14) | 1.64 (0.88, 3.06) | -0.26 (-0.99, 0.47) |
| Richer | 1.17 (0.82, 1.68) | -0.27 (-0.82, 0.27) | 1.51 (0.81, 2.80) | -0.48 (-1.23, 0.27) |
| Richest | 1.30 (0.91, 1.86) | -0.52 (-1.06, 0.01) | 2.21* (1.16, 4.20) | -0.76 (-1.63, 0.11) |
| **Religion** |  |  |  |  |
| Hindu | Ref. | Ref. | Ref. | Ref. |
| Muslim | 0.99 (0.69, 1.42) | -0.29 (-0.97, 0.40) | 0.75 (0.44, 1.30) | -0.41 (-1.04, 0.21) |
| Christian | 0.70 (0.21, 2.28) | 0.53 (-0.43, 1.49) | 0.52 (0.19, 1.42) | 0.78 (-0.48, 2.05) |
| Others | 1.33 (0.79, 2.24) | 0.89* (0.20, 1.58) | 0.31* (0.11, 0.83) | -0.60 (-1.69, 0.49) |
| **Caste** |  |  |  |  |
| Scheduled Caste | Ref. | Ref. | Ref. | Ref. |
| Scheduled Tribe | 0.46** (0.28, 0.74) | 1.16*** (0.48, 1.84) | 0.78 (0.31, 1.95) | 0.33 (-1.70, 2.35) |
| Other Backward Class | 0.96 (0.71, 1.30) | -0.45 (-0.90, 0.00) | 0.85 (0.50, 1.46) | -0.28 (-1.10, 0.54) |
| Others | 0.95 (0.68, 1.32) | -0.26 (-0.78, 0.26) | 0.63 (0.36, 1.10) | -0.21 (-1.02, 0.59) |
| **Region** |  |  |  |  |
| North | Ref. | Ref. | Ref. | Ref. |
| Central | 2.33*** (1.66, 3.27) | 0.08 (-0.46, 0.63) | 1.18 (0.67, 2.07) | -0.28 (-1.03, 0.47) |
| East | 1.09 (0.77, 1.54) | 0.22 (-0.23, 0.67) | 0.83 (0.46, 1.49) | -1.10** (-1.80, -0.41) |
| Northeast | 1.16 (0.64, 2.11) | -0.07 (-0.71, 0.58) | 0.60 (0.19, 1.88) | -0.65 (-1.62, 0.33) |
| West | 0.64* (0.42, 0.96) | -0.08 (-0.58, 0.43) | 0.49* (0.26, 0.90) | -0.41 (-1.19, 0.37) |
| South | 1.02 (0.64, 1.62) | 1.30*** (0.72, 1.87) | 0.86 (0.46, 1.61) | 0.64 (-0.07, 1.34) |

**Table-S2b** Multivariate logistic and linear regression estimates of depression and worse cognitive function by widowhood status/duration and other background characteristics among **women**, stratified by rural/urban place of residence, LASI, 2017-2019

| **Background characteristic** | Rural | | Urban | |
| --- | --- | --- | --- | --- |
|  | Depression | Worse cognitive function | Depression | Worse cognitive function |
| **Widowhood duration** |  |  |  |  |
| Currently married | Ref. | Ref. | Ref. | Ref. |
| Widows (0-9 years) | 1.55** (1.19, 2.02) | 0.47* (0.04, 0.90) | 1.71* (1.10, 2.67) | 0.74* (0.10, 1.37) |
| Widows (10-19 years) | 1.17 (0.83, 1.65) | 0.57* (0.05, 1.09) | 1.21 (0.57, 2.59) | 0.76 (-0.11, 1.63) |
| Widows (20+ years) | 1.04 (0.77, 1.41) | 0.70** (0.24, 1.16) | 1.39 (0.83, 2.33) | 1.21** (0.39, 2.04) |
| **Age** |  |  |  |  |
| 60-69 years | Ref. | Ref. | Ref. | Ref. |
| 70-79 years | 0.94 (0.74, 1.19) | 1.00*** (0.61, 1.40) | 0.82 (0.55, 1.23) | 1.37*** (0.78, 1.96) |
| > 80 years | 1.01 (0.69, 1.46) | 2.36*** (1.76, 2.95) | 0.81 (0.43, 1.54) | 2.96*** (1.68, 4.23) |
| **Education** |  |  |  |  |
| No formal education | Ref. | Ref. | Ref. | Ref. |
| Primary not completed | 1.14 (0.76, 1.69) | -2.95*** (-3.52, -2.37) | 1.37 (0.78, 2.42) | -3.34*** (-4.13, -2.54) |
| Primary | 1.46 (0.98, 2.17) | -5.83*** (-6.47, -5.19) | 1.17 (0.67, 2.05) | -5.26*** (-6.10, -4.41) |
| Secondary/higher | 1.26 (0.72, 2.21) | -8.84*** (-9.59, -8.08) | 1.02 (0.56, 1.87) | -7.69*** (-8.44, -6.94) |
| **Multigenerational living** |  |  |  |  |
| Yes | Ref. | Ref. | Ref. | Ref. |
| No | 1.23 (0.99, 1.52) | 0.31 (-0.03, 0.66) | 1.70* (1.06, 2.71) | 0.62 (-0.05, 1.30) |
| **Working status** |  |  |  |  |
| Currently working | Ref. | Ref. | Ref. | Ref. |
| Retired | 1.52** (1.17, 1.97) | -0.21 (-0.61, 0.18) | 1.26 (0.80, 1.97) | -0.38 (-0.96, 0.20) |
| Never worked | 1.71*** (1.28, 2.30) | -0.93*** (-1.35, -0.51) | 2.17* (1.02, 4.62) | -0.36 (-1.22, 0.50) |
| Currently not working | 1.94 (0.90, 4.17) | -2.01** (-3.23, -0.78) | 1.85 (0.79, 4.32) | -2.32*** (-3.55, -1.09) |
| **Social participation** |  |  |  |  |
| No | Ref. | Ref. | Ref. | Ref. |
| Yes | 1.28 (0.80, 2.06) | -0.76* (-1.41, -0.10) | 0.64 (0.20, 2.07) | -0.86 (-1.90, 0.19) |
| **Life satisfaction** |  |  |  |  |
| Low | Ref. | Ref. | Ref. | Ref. |
| Medium | 0.50*** (0.38, 0.64) | -0.78*** (-1.18, -0.38) | 0.69 (0.43, 1.10) | -0.67 (-1.49, 0.15) |
| High | 0.61*** (0.47, 0.78) | -0.86*** (-1.24, -0.49) | 0.48** (0.28, 0.80) | -0.99*** (-1.54, -0.45) |
| **Self-rated health** |  |  |  |  |
| Good | Ref. | Ref. | Ref. | Ref. |
| Poor | 2.14*** (1.71, 2.69) | 0.65*** (0.26, 1.04) | 1.95*** (1.34, 2.83) | 0.31 (-0.26, 0.87) |
| **Difficulty with ADL** |  |  |  |  |
| No | Ref. | Ref. | Ref. | Ref. |
| Yes | 1.44** (1.14, 1.83) | 0.47* (0.02, 0.93) | 1.51 (1.00, 2.27) | 0.49 (-0.11, 1.10) |
| **Difficulty with IADL** |  |  |  |  |
| No | Ref. | Ref. | Ref. | Ref. |
| Yes | 1.71*** (1.34, 2.18) | 0.73*** (0.40, 1.07) | 1.63* (1.08, 2.45) | 1.43*** (0.83, 2.04) |
| **Insomnia symptoms** |  |  |  |  |
| No | Ref. | Ref. | Ref. | Ref. |
| Yes | 1.99*** (1.61, 2.46) | 0.17 (-0.15, 0.49) | 2.47*** (1.61, 3.81) | -0.12 (-0.62, 0.38) |
| **MPCE quintile** |  |  |  |  |
| Poorest | Ref. | Ref. | Ref. | Ref. |
| Poorer | 1.08 (0.81, 1.45) | -0.24 (-0.73, 0.26) | 0.84 (0.50, 1.40) | -0.46 (-1.18, 0.27) |
| Middle | 1.04 (0.75, 1.44) | -0.52* (-1.02, -0.02) | 0.55 (0.26, 1.18) | -1.05** (-1.81, -0.29) |
| Richer | 1.16 (0.83, 1.60) | -0.87*** (-1.37, -0.38) | 0.92 (0.55, 1.56) | -0.49 (-1.26, 0.28) |
| Richest | 1.49* (1.09, 2.04) | -1.11*** (-1.65, -0.58) | 0.69 (0.37, 1.26) | -0.58 (-1.54, 0.38) |
| **Religion** |  |  |  |  |
| Hindu | Ref. | Ref. | Ref. | Ref. |
| Muslim | 1.32 (0.87, 1.98) | 0.48 (-0.06, 1.01) | 0.69 (0.42, 1.15) | -0.12 (-0.84, 0.61) |
| Christian | 2.38** (1.33, 4.23) | -0.03 (-0.93, 0.87) | 1.53 (0.55, 4.27) | -0.30 (-1.51, 0.90) |
| Others | 1.26 (0.76, 2.10) | -0.39 (-1.06, 0.29) | 1.02 (0.42, 2.44) | -0.78 (-1.94, 0.38) |
| **Caste** |  |  |  |  |
| Scheduled Caste | Ref. | Ref. | Ref. | Ref. |
| Scheduled Tribe | 0.55** (0.36, 0.83) | 0.77** (0.21, 1.33) | 0.51 (0.17, 1.58) | -0.15 (-1.65, 1.35) |
| Other Backward Class | 1.36* (1.04, 1.78) | -0.78*** (-1.20, -0.35) | 2.01* (1.04, 3.88) | 0.09 (-0.79, 0.97) |
| Others | 1.05 (0.76, 1.45) | -0.76** (-1.21, -0.31) | 1.50 (0.67, 3.36) | 0.14 (-0.71, 1.00) |
| **Region** |  |  |  |  |
| North | Ref. | Ref. | Ref. | Ref. |
| Central | 2.70*** (1.94, 3.76) | -0.26 (-0.71, 0.19) | 2.51** (1.42, 4.46) | -1.05** (-1.82, -0.28) |
| East | 0.97 (0.70, 1.34) | -0.29 (-0.75, 0.16) | 1.23 (0.72, 2.09) | -0.90* (-1.65, -0.15) |
| Northeast | 0.84 (0.52, 1.33) | -0.26 (-0.86, 0.35) | 1.74 (0.73, 4.10) | -0.59 (-1.80, 0.62) |
| West | 0.58** (0.39, 0.86) | -1.14*** (-1.62, -0.66) | 0.46** (0.27, 0.77) | -1.64*** (-2.45, -0.82) |
| South | 1.31 (0.91, 1.91) | 1.45*** (0.89, 2.01) | 1.25 (0.68, 2.28) | 0.28 (-0.42, 0.98) |

Figure S2: Interaction effect of widowhood status/duration and rural/urban place of residence on depression among men and women

Figure S3: Interaction effect of widowhood status/duration and rural/urban place of residence on cognitive function among men and women

**Table-S3a** Multivariate logistic and linear regression estimates of depression and worse cognitive function by widowhood status/duration and other background characteristics among **men**, stratified by multigenerational living status, LASI, 2017-2019

| **Background characteristic** | **Non-multigenerational living** | | **Multigenerational living** | |
| --- | --- | --- | --- | --- |
|  | **Depression, AOR (95%CI)** | **Worse cognitive function, b (95%CI)** | **Depression, AOR (95%CI)** | **Worse cognitive function, b (95%CI)** |
| **Widowhood duration** |  |  |  |  |
| Currently married | Ref. | Ref. | Ref. | Ref. |
| Recent widows (0-9 years) | 1.72 (0.98, 3.00) | -0.43 (-1.52, 0.66) | 1.60* (1.09, 2.35) | 1.13*** (0.57, 1.69) |
| Mid-term widows (10-19 years) | 1.48 (0.55, 3.97) | 0.48 (-0.72, 1.67) | 0.75 (0.40, 1.38) | -0.27 (-1.03, 0.48) |
| Long-term widows (20+ years) | 0.62 (0.24, 1.56) | -0.28 (-2.46, 1.90) | 0.67 (0.36, 1.25) | 1.16** (0.36, 1.97) |
| **Age** |  |  |  |  |
| 60-69 years | Ref. | Ref. | Ref. | Ref. |
| 70-79 years | 0.66* (0.45, 0.97) | 1.23*** (0.58, 1.88) | 0.65** (0.50, 0.85) | 0.85*** (0.50, 1.20) |
| > 80 years | 0.72 (0.40, 1.31) | 2.31*** (1.17, 3.44) | 0.67 (0.38, 1.18) | 1.64*** (0.96, 2.31) |
| **Education** |  |  |  |  |
| No formal education | Ref. | Ref. | Ref. | Ref. |
| Primary not completed | 1.23 (0.72, 2.10) | -3.51*** (-4.30, -2.72) | 1.23 (0.80, 1.91) | -3.34*** (-3.92, -2.76) |
| Primary | 1.29 (0.79, 2.12) | -4.48*** (-5.36, -3.60) | 1.08 (0.77, 1.50) | -4.71*** (-5.17, -4.26) |
| Secondary/higher | 1.46 (0.87, 2.44) | -6.89*** (-7.63, -6.15) | 0.90 (0.66, 1.22) | -6.90*** (-7.32, -6.48) |
| **Working status** |  |  |  |  |
| Currently working | Ref. | Ref. | Ref. | Ref. |
| Retired | 0.88 (0.40, 1.92) | -1.85* (-3.59, -0.11) | 0.91 (0.48, 1.73) | 0.01 (-0.88, 0.90) |
| Never worked | 0.99 (0.45, 2.18) | -1.49 (-3.17, 0.18) | 0.80 (0.42, 1.52) | -0.27 (-1.17, 0.63) |
| Currently not working | 1.15 (0.45, 2.93) | -2.32* (-4.10, -0.53) | 0.77 (0.39, 1.53) | -0.83 (-1.75, 0.09) |
| **Social participation** |  |  |  |  |
| No | Ref. | Ref. | Ref. | Ref. |
| Yes | 0.90 (0.37, 2.24) | -0.75 (-1.50, 0.00) | 0.71 (0.44, 1.15) | -0.56* (-1.02, -0.09) |
| **Life satisfaction** |  |  |  |  |
| Low | Ref. | Ref. | Ref. | Ref. |
| Medium | 0.47** (0.30, 0.74) | -0.58 (-1.25, 0.09) | 0.69* (0.49, 0.97) | -0.16 (-0.63, 0.31) |
| High | 0.37*** (0.24, 0.57) | -1.11*** (-1.73, -0.48) | 0.46*** (0.35, 0.60) | -0.12 (-0.53, 0.29) |
| **Self-rated health** |  |  |  |  |
| Good | Ref. | Ref. | Ref. | Ref. |
| Poor | 2.13*** (1.45, 3.12) | 0.47 (-0.21, 1.14) | 2.26*** (1.73, 2.95) | 0.97*** (0.58, 1.37) |
| **Difficulty with ADL** |  |  |  |  |
| No | Ref. | Ref. | Ref. | Ref. |
| Yes | 1.39 (0.95, 2.05) | 0.74 (-0.04, 1.52) | 1.91*** (1.30, 2.79) | 0.23 (-0.30, 0.76) |
| **Difficulty with IADL** |  |  |  |  |
| No | Ref. | Ref. | Ref. | Ref. |
| Yes | 1.90*** (1.32, 2.74) | 0.44 (-0.21, 1.10) | 1.39 (0.99, 1.97) | 1.58*** (1.17, 2.00) |
| **Insomnia symptoms** |  |  |  |  |
| No | Ref. | Ref. | Ref. | Ref. |
| Yes | 1.78** (1.26, 2.53) | 0.40 (-0.17, 0.97) | 1.92*** (1.51, 2.44) | -0.07 (-0.41, 0.26) |
| **MPCE quintile** |  |  |  |  |
| Poorest | Ref. | Ref. | Ref. | Ref. |
| Poorer | 0.51* (0.27, 0.97) | -0.54 (-1.45, 0.36) | 0.94 (0.67, 1.32) | 0.28 (-0.18, 0.74) |
| Middle | 0.89 (0.50, 1.58) | -1.19** (-2.03, -0.35) | 1.34 (0.88, 2.05) | -0.06 (-0.55, 0.43) |
| Richer | 0.72 (0.41, 1.29) | -1.41** (-2.35, -0.48) | 1.58* (1.09, 2.30) | -0.01 (-0.50, 0.49) |
| Richest | 0.89 (0.50, 1.55) | -1.35** (-2.26, -0.44) | 1.80** (1.24, 2.63) | -0.41 (-0.94, 0.13) |
| **Religion** |  |  |  |  |
| Hindu | Ref. | Ref. | Ref. | Ref. |
| Muslim | 1.18 (0.64, 2.18) | 0.37 (-0.91, 1.64) | 0.85 (0.60, 1.20) | -0.53* (-1.04, -0.02) |
| Christian | 0.86 (0.18, 4.15) | 1.01 (-0.26, 2.27) | 0.48 (0.21, 1.08) | 0.36 (-0.62, 1.34) |
| Others | 1.92 (0.80, 4.60) | 1.21 (-0.08, 2.50) | 0.68 (0.41, 1.14) | 0.19 (-0.49, 0.87) |
| **Caste** |  |  |  |  |
| Scheduled Caste | Ref. | Ref. | Ref. | Ref. |
| Scheduled Tribe | 0.40 (0.15, 1.03) | 1.43* (0.24, 2.62) | 0.56* (0.34, 0.90) | 1.10** (0.36, 1.84) |
| Other Backward Class | 1.04 (0.65, 1.64) | -0.51 (-1.23, 0.22) | 0.89 (0.64, 1.24) | -0.32 (-0.79, 0.15) |
| Others | 1.18 (0.68, 2.02) | -0.22 (-1.07, 0.63) | 0.72 (0.51, 1.02) | -0.22 (-0.72, 0.28) |
| **Place of residence** |  |  |  |  |
| Rural | Ref. | Ref. | Ref. | Ref. |
| Urban | 1.00 (0.65, 1.54) | -0.77* (-1.49, -0.05) | 1.00 (0.76, 1.33) | -1.35*** (-1.71, -1.00) |
| **Region** |  |  |  |  |
| North | Ref. | Ref. | Ref. | Ref. |
| Central | 3.43*** (1.87, 6.29) | -0.10 (-1.04, 0.84) | 1.61** (1.15, 2.26) | -0.08 (-0.58, 0.43) |
| East | 1.92* (1.03, 3.59) | -0.08 (-0.86, 0.70) | 0.75 (0.53, 1.07) | -0.11 (-0.55, 0.33) |
| Northeast | 1.23 (0.40, 3.80) | -0.64 (-1.93, 0.64) | 0.89 (0.49, 1.61) | -0.22 (-0.83, 0.38) |
| West | 0.87 (0.44, 1.71) | -0.00 (-0.76, 0.76) | 0.52** (0.35, 0.79) | -0.25 (-0.78, 0.27) |
| South | 1.56 (0.73, 3.33) | 0.83 (-0.11, 1.78) | 0.82 (0.53, 1.25) | 1.13*** (0.63, 1.64) |

**Table-S3b** Multivariate logistic and linear regression estimates of depression and worse cognitive function by widowhood status/duration and other background characteristics among **women**, stratified by multigenerational living status, LASI, 2017-2019

| **Background characteristic** | **Non-multigenerational living** | | **Multigenerational living** | |
| --- | --- | --- | --- | --- |
|  | **Depression, AOR (95%CI)** | **Worse cognitive function, b (95%CI)** | **Depression, AOR (95%CI)** | **Worse cognitive function, b (95%CI)** |
| **Widowhood duration** |  |  |  |  |
| Currently married | Ref. | Ref. | Ref. | Ref. |
| Recent widows (0-9 years) | 1.99*** (1.34, 2.97) | -0.11 (-0.80, 0.59) | 1.38* (1.04, 1.82) | 0.86*** (0.45, 1.26) |
| Mid-term widows (10-19 years) | 1.03 (0.53, 1.99) | 0.10 (-0.63, 0.83) | 1.20 (0.86, 1.69) | 0.81** (0.21, 1.42) |
| Long-term widows (20+ years) | 1.16 (0.74, 1.83) | 0.38 (-0.40, 1.16) | 1.04 (0.75, 1.43) | 1.06*** (0.55, 1.57) |
| **Age** |  |  |  |  |
| 60-69 years | Ref. | Ref. | Ref. | Ref. |
| 70-79 years | 0.88 (0.62, 1.25) | 0.98** (0.35, 1.61) | 0.91 (0.70, 1.17) | 1.29*** (0.88, 1.70) |
| > 80 years | 1.17 (0.71, 1.93) | 2.75*** (1.75, 3.76) | 0.84 (0.54, 1.30) | 2.53*** (1.82, 3.24) |
| **Education** |  |  |  |  |
| No formal education | Ref. | Ref. | Ref. | Ref. |
| Primary not completed | 1.42 (0.79, 2.54) | -3.25*** (-4.27, -2.23) | 1.20 (0.81, 1.76) | -3.11*** (-3.63, -2.58) |
| Primary | 1.46 (0.80, 2.66) | -5.71*** (-6.96, -4.45) | 1.25 (0.84, 1.84) | -5.52*** (-6.09, -4.95) |
| Secondary/higher | 1.44 (0.67, 3.08) | -8.89*** (-9.93, -7.84) | 0.94 (0.59, 1.49) | -7.61*** (-8.28, -6.93) |
| **Working status** |  |  |  |  |
| Currently working | Ref. | Ref. | Ref. | Ref. |
| Retired | 1.10 (0.75, 1.62) | -0.37 (-1.00, 0.25) | 1.70*** (1.30, 2.23) | -0.26 (-0.65, 0.13) |
| Never worked | 1.69* (1.04, 2.74) | -1.08** (-1.76, -0.40) | 1.84*** (1.35, 2.50) | -0.64** (-1.10, -0.19) |
| Currently not working | 2.64* (1.15, 6.07) | -1.73* (-3.10, -0.37) | 1.36 (0.60, 3.09) | -2.61*** (-3.70, -1.52) |
| **Social participation** |  |  |  |  |
| No | Ref. | Ref. | Ref. | Ref. |
| Yes | 0.73 (0.33, 1.64) | -0.41 (-1.43, 0.60) | 1.43 (0.85, 2.42) | -1.01** (-1.69, -0.34) |
| **Life satisfaction** |  |  |  |  |
| Low | Ref. | Ref. | Ref. | Ref. |
| Medium | 0.55** (0.38, 0.81) | -0.63 (-1.35, 0.09) | 0.52*** (0.39, 0.68) | -0.82*** (-1.23, -0.41) |
| High | 0.43*** (0.28, 0.65) | -0.95** (-1.54, -0.37) | 0.64** (0.49, 0.84) | -0.87*** (-1.25, -0.48) |
| **Self-rated health** |  |  |  |  |
| Good | Ref. | Ref. | Ref. | Ref. |
| Poor | 2.49*** (1.79, 3.47) | 0.53 (-0.08, 1.13) | 1.92*** (1.51, 2.44) | 0.60** (0.23, 0.98) |
| **Difficulty with ADL** |  |  |  |  |
| No | Ref. | Ref. | Ref. | Ref. |
| Yes | 1.18 (0.83, 1.67) | 0.45 (-0.22, 1.12) | 1.64*** (1.28, 2.11) | 0.47* (0.02, 0.91) |
| **Difficulty with IADL** |  |  |  |  |
| No | Ref. | Ref. | Ref. | Ref. |
| Yes | 1.72** (1.19, 2.49) | 0.65* (0.08, 1.23) | 1.70*** (1.31, 2.20) | 1.11*** (0.74, 1.48) |
| **Insomnia symptoms** |  |  |  |  |
| No | Ref. | Ref. | Ref. | Ref. |
| Yes | 2.22*** (1.62, 3.04) | 0.26 (-0.25, 0.77) | 2.06*** (1.63, 2.60) | 0.01 (-0.32, 0.34) |
| **MPCE quintile** |  |  |  |  |
| Poorest | Ref. | Ref. | Ref. | Ref. |
| Poorer | 0.94 (0.57, 1.54) | -0.10 (-0.93, 0.74) | 1.07 (0.80, 1.43) | -0.44 (-0.93, 0.04) |
| Middle | 0.94 (0.56, 1.60) | -0.77 (-1.58, 0.05) | 0.91 (0.65, 1.29) | -0.65** (-1.13, -0.17) |
| Richer | 1.05 (0.67, 1.64) | -0.86* (-1.59, -0.12) | 1.16 (0.80, 1.69) | -0.70* (-1.24, -0.15) |
| Richest | 1.00 (0.62, 1.61) | -1.10* (-2.00, -0.19) | 1.56** (1.11, 2.18) | -0.84** (-1.44, -0.23) |
| **Religion** |  |  |  |  |
| Hindu | Ref. | Ref. | Ref. | Ref. |
| Muslim | 1.09 (0.64, 1.86) | 1.00* (0.15, 1.84) | 1.12 (0.74, 1.71) | -0.01 (-0.50, 0.48) |
| Christian | 3.29*** (1.62, 6.67) | -0.65 (-1.99, 0.68) | 1.25 (0.57, 2.70) | 0.24 (-0.59, 1.07) |
| Others | 1.80 (0.84, 3.87) | -0.68 (-2.01, 0.65) | 1.02 (0.61, 1.68) | -0.60 (-1.26, 0.07) |
| **Caste** |  |  |  |  |
| Scheduled Caste | Ref. | Ref. | Ref. | Ref. |
| Scheduled Tribe | 0.72 (0.38, 1.38) | 1.06* (0.15, 1.98) | 0.48** (0.29, 0.77) | 0.46 (-0.21, 1.13) |
| Other Backward Class | 1.75* (1.13, 2.71) | -0.56 (-1.25, 0.13) | 1.29 (0.97, 1.73) | -0.59* (-1.07, -0.11) |
| Others | 1.49 (0.85, 2.62) | -0.50 (-1.28, 0.29) | 0.98 (0.70, 1.37) | -0.57* (-1.06, -0.09) |
| **Place of residence** |  |  |  |  |
| Rural | Ref. | Ref. | Ref. | Ref. |
| Urban | 0.74 (0.42, 1.29) | -0.93* (-1.67, -0.19) | 0.79 (0.60, 1.03) | -1.64*** (-2.01, -1.26) |
| **Region** |  |  |  |  |
| North | Ref. | Ref. | Ref. | Ref. |
| Central | 2.47** (1.43, 4.28) | 0.11 (-0.67, 0.90) | 2.91*** (2.08, 4.06) | -0.76*** (-1.20, -0.32) |
| East | 1.00 (0.58, 1.71) | -0.40 (-1.21, 0.40) | 1.08 (0.79, 1.49) | -0.48* (-0.93, -0.02) |
| Northeast | 1.51 (0.72, 3.17) | 0.15 (-0.90, 1.21) | 0.86 (0.53, 1.41) | -0.56 (-1.19, 0.06) |
| West | 0.54* (0.30, 0.95) | -1.15** (-1.92, -0.38) | 0.56** (0.38, 0.82) | -1.21*** (-1.77, -0.66) |
| South | 1.43 (0.81, 2.55) | 1.40** (0.52, 2.27) | 1.17 (0.80, 1.70) | 0.83** (0.32, 1.33) |

Figure S4: Interaction effect of widowhood status/duration and multigenerational living status on depression among men and women

Figure S5: Interaction effect of widowhood status/duration and multigenerational living status on cognitive function among men and women
